# Lactate promotes macrophage HMGB1 lactylation, acetylation, and exosomal release in polymicrobial sepsis

**DOI:** 10.1038/s41418-021-00841-9

**Published:** 2021-08-06

**Authors:** Kun Yang, Min Fan, Xiaohui Wang, Jingjing Xu, Yana Wang, Fei Tu, P. Spencer Gill, Tuanzhu Ha, Li Liu, David L. Williams, Chuanfu Li

**Affiliations:** 1 Department of Surgery, James H. Quillen College of Medicine, East Tennessee State University, Johnson City, TN USA; 2 Center of Excellence in Inflammation, Infectious Disease and Immunity, East Tennessee State University, Johnson City, TN USA; 3 Department of Geriatrics, The First Affiliated Hospital of Nanjing Medical University, Nanjing, Jiangsu China

**Keywords:** Infectious diseases, Signal transduction, Epigenetics

## Abstract

High circulating levels of lactate and high mobility group box-1 (HMGB1) are associated with the severity and mortality of sepsis. However, it is unclear whether lactate could promote HMGB1 release during sepsis. The present study demonstrated a novel role of lactate in HMGB1 lactylation and acetylation in macrophages during polymicrobial sepsis. We found that macrophages can uptake extracellular lactate via monocarboxylate transporters (MCTs) to promote HMGB1 lactylation via a p300/CBP-dependent mechanism. We also observed that lactate stimulates HMGB1 acetylation by Hippo/YAP-mediated suppression of deacetylase SIRT1 and β-arrestin2-mediated recruitment of acetylases p300/CBP to the nucleus via G protein-coupled receptor 81 (GPR81). The lactylated/acetylated HMGB1 is released from macrophages via exosome secretion which increases endothelium permeability. In vivo reduction of lactate production and/or inhibition of GPR81-mediated signaling decreases circulating exosomal HMGB1 levels and improves survival outcome in polymicrobial sepsis. Our results provide the basis for targeting lactate/lactate-associated signaling to combat sepsis.

## Introduction

Sepsis is a life-threatening disease that is characterized by organ dysfunction and dysregulated host innate and inflammatory responses to the infection [1]. Serum lactate has been recognized as a biomarker in sepsis prognosis and elevated serum lactate levels are positively correlated with sepsis mortality [2]. The recent Sepsis-3 guidelines recommend that persistent serum lactate levels >2 mmol/L, despite adequate fluid resuscitation, should be included as a new criterion when clinically defining septic shock [3]. However, whether lactate can play a causal role in sepsis remains largely unknown.

HMGB1 is a ubiquitous nuclear protein that can be released by activated macrophages to orchestrate inflammatory responses [4–6]. Clinical evidence has revealed that the levels of circulating HMGB1 are markedly elevated and positively correlated with sepsis severity and mortality [7–9]. Notably, a recent single-institution study, including 218 critically ill patients (145 with sepsis and 73 without sepsis), revealed that blood HMGB1 levels positively correlated with blood lactate levels (*r* = 0.144, *P* = 0.035), suggesting that lactate could regulate HMGB1 release in sepsis [10]. Previous studies have shown that post-translational modification (i.e., acetylation, phosphorylation, and methylation) of HMGB1 at the region close to or within the nuclear localization sequences (NLSs) could induce its translocation to the cytoplasm, leading to subsequent release of HMGB1 during inflammation [11–13]. Recently, Zhang et al. reported a novel function of glycolysis-derived lactate in macrophages whereby it is utilized in modulating nuclear histones through the addition of lactyl groups to the lysine (K) residues of histones [14]. This process is termed as “lactylation.”’ However, it remains unknown whether lactate could promote HMGB1 lactylation and release. In addition, while it is reported that aerobic glycolysis can stimulate HMGB1 acetylation in macrophages [15], little is known about the roles of lactate in HMGB1 acetylation and release during sepsis.

The present study investigated whether lactate could promote HMGB1 lactylation and acetylation in macrophages during polymicrobial sepsis. We observed that serum exosomes contain high levels of HMGB1, which are positively correlated with serum lactate levels in polymicrobial septic mice. We demonstrated a novel role of lactate in promoting HMGB1 lactylation and acetylation and, resulting in enhanced HMGB1 release via exosome secretion from macrophages. Furthermore, our data indicated that such macrophage-derived exosomal HMGB1 could markedly increase endothelial cell permeability. In comparison, pharmacological inhibition of lactate production and/or lactate receptor GPR81-mediated signaling decreases circulating exosomal HMGB1 levels, which highlights lactate/lactate-associated signaling as a promising drug target in sepsis.

## Materials and methods

### Animal information

YAP^flox^/TAZ^flox^ (JAX 030532), Lyz2-Cre (JAX 004781), and wild-type C57BL/6 J (JAX 000664) mice were purchased from Jackson Laboratory (Indianapolis, IN). Macrophage-specific YAP knockout mice were generated using the Cre/Loxp system. In general, *YAP*^flox/flox^ mice were crossbred with lysozyme2 (Lyz2)-Cre mice. Genotypes for the macrophages specific deletion of YAP were confirmed by PCR analysis using the following primers: YAP F: 5′ CCC AAA TTT GAA TCA TTG GGG TCT TTG C 3′; YAP R1: AAC AAA ACC TGG GGA ACG ACT GGG CAC T 3′; YAP R2: GTG CAT AGC TGC ATA ACT TCG TAT AAT GT 3′. Four hundred and ten base pairs for flox allele (F-R1), 315 bp for wild-type allele (F-R1), and 230 bp floxed (deleted) allele (F-R2). Cre gene expression was also examined by PCR. All mice were maintained and bred at the Division of Laboratory Animal Resources at East Tennessee State University (ETSU). All experimental procedures were performed in accordance with the Guide for the Care and Use of Laboratory Animals published by the National Institutes of Health (NIH Publication, 8th Edition, 2011) and approved by the ETSU Committee on Animal Care. Age- and sex-matched 8–12 weeks mice were used for all experiments. Mice were randomly allocated into each treatment group. Investigators were blinded to group allocation during data collection and analysis.

### Cecal ligation and puncture (CLP) polymicrobial sepsis model

Polymicrobial sepsis was induced by CLP as described previously [16]. Briefly, mice were anesthetized by 5.0% isoflurane. After anesthesia, the abdomen was shaved, and the cecum was exposed through a 1 cm midline incision. The cecum was ligated between 3rd and 4th vascular arcade with a 4-0 silk suture and punctured with a 25-gauge needle. Sham surgically operated mice were served as sham controls. Following surgery, a single dose of resuscitative fluid was administrated by subcutaneous injection. Recovery was facilitated by placing mice on a heated pad. Lactic acid (pH 6.8, 0.5 g/kg body weight) was intraperitoneally injected 6 h after CLP or sham surgical operation. Terminal collection of blood was done via the vena cava. Serum was prepared as described elsewhere and stored at −80 °C for further experiments.

### Cell culture

Murine RAW 264.7 macrophages were purchased from ATCC and cultured in DMEM supplemented with penicillin (100 U/mL) and streptomycin (100 ng/mL), and 10% FBS. Macrophages were treated with 10 mM L-lactic acid (Sigma) or sodium L-lactate (Sigma) for 6–24 h. In a separate experiment, macrophages were treated with lactic acid (10 mM) for 30 min followed by LPS (500 ng/mL) stimulation for 6 h. To inhibit lactate production, macrophages were treated with oxamate (20 mM) for 2 h followed by LPS (500 ng/mL) stimulation for 6 h. 3-hydroxy-butyrate (3-OBA, Sigma), Compound C (CC, Selleckchem), C646 (Sigma), Selisstat (EX527, Selleckchem), and SRT2183 (Selleckchem) were used for in vitro experiments. For hypoxia condition, macrophages were incubated at 37 °C with 5% CO_2_ and 0.1% O_2_ in a hypoxia chamber (Pro-Ox Model C21, BioSpherix Ltd., Redfield NY) for 24 h.

### Isolation of primary peritoneal macrophages

Peritoneal macrophages were collected as described previously [17]. Briefly, THE inner skin lining the peritoneal cavity was exposed by cutting the outer skin of the peritoneum. Five microliter PBS was injected into the peritoneal cavity using a G27 needle. To increase the yield of peritoneal macrophages, attached macrophages were dislodged by gently massaging the peritoneum. Fluid was collected and centrifuged 500 g for 5 min. The purity of THE macrophage population was confirmed by flow cytometry with anti-CD11b and anti-F4/80 antibodies.

### Isolation and culturing of primary bone marrow-derived macrophages (BMDMs)

Bone marrow was obtained by flushing the femur and tibia with DMEM supplemented with penicillin (100 U/mL) and streptomycin (100 ng/mL), and 10% fetal bovine serum (FBS). A single-cell suspension of bone marrow cells was prepared by passing the bone marrow through a 70-μm cell strainer. Bone marrow cells were then differentiated to BMDMs by culturing for 7 days in DMEM (100 U/mL penicillin, 100 ng/mL streptomycin, and 10% FBS) supplemented with 10% L929-conditioned medium. The presence of macrophage marker F4/80 was confirmed by immunofluorescent staining.

### Cell transfection and transduction

siRNAs and adenovirus recombinant were listed in Table S1. Specifically, RAW 264.7 macrophages were transfected with 40 nM siRNAs using Lipofectamine 3000 (Invitrogen, CA) according to the manufacturer’s protocol. After transfection for 24 h, cells were either harvested for analysis or used for the following experiments. To overexpress YAP in macrophages, RAW 264.7 cells were transduced with either Ad-YAP or Ad-GFP for 24–36 h. Similarly, SIRT1 overexpression was achieved by transducing RAW 264.7 cells with Ad-SIRT1 or Ad-GFP for 24–36 h. After transduction, cells were either harvested for analysis or used for the following experiments.

### Flow cytometry

Expression of CD11b and F4/80 was examined by flow cytometry to determine the purity of isolated peritoneal macrophages and BMDMs. Cells were incubated with human IgG to block Fc-receptors for 15 min before staining with specific single-color antibodies overnight at 4 °C. After washing, cells were analyzed by FACSfortessa flow cytometer (Becton Dickinson). The cell death and apoptosis were evaluated using FITC Annexin V Apoptosis Decection Kit with PI (Biolegend) and analyzed by FACSfortessa flow cytometer (Becton Dickinson). Results were analyzed using the FlowJo software.

### Exosome enrichment and characterization

Exosomes were prepared using a polyethylene glycol (PEG)-based method [18]. To isolate exosomes from the conditioned medium of macrophages, cells were cultured in DMEM supplemented with 10% exosome-depleted FBS (ThermoFisher) for 24 h and exosome-containing supernatant was collected. Cell debris was removed by centrifuge at 1500 g for 10 min. Ten mililiter of exosome-containing supernatant was mixed with 5 mL 34% PEG 6000 (Sigma) solution. To isolate exosomes from serum, 50 μL serum was diluted in 150 μL PBS followed by mixing with 100 μL 34% PEG solution. After intensive mixing, the mixture was incubated overnight (at least 14 h) at 4 °C followed by centrifugation at 3000 g for 1 h. The supernatant was completely removed and the remaining pellet at the bottom of the tube was resuspended in RIPA buffer for 3 min on ice to prepare exosomal lysate or resuspended in PBS for following experiments. The quality of exosomes was confirmed by dynamic light scattering using a particle and molecule size analyzer (ZetasizerNano ZS, Malvern Instruments) according to the manufacturer’s instruction. The presence of exosome markers, including HSP70, CD63, Alix, and the absence of calnexin was confirmed by western blot.

### Enzyme-linked immunosorbent assay (ELISA)

Serum exosomes were depleted using Total Exosome Isolation Reagent (Thermofisher) according to manufacturer’s instruction. HMGB1 levels in exosome-depleted and exosome-undepleted serum were measured by mouse HMGB1 ELISA kit (Abclonal).

### Western blot

Cell or exosome lysates were prepared in ice-cold cell fractionation kit (Abcam) or RIPA buffer containing protease inhibitor (ThermoFisher). Lysates were centrifuged at 14,000 g for 10 min at 4 °C. Protein amounts were quantified with Pierce BCA protein assay kit (ThermoFisher). Western blot was carried out using standard techniques with antibodies outlined in Supplementary Table S1. Briefly, cellular or exosomal proteins were separated by sodium dodecyl sulphate–polyacrylamide gel electrophoresis and transferred onto nitrocellulose blotting membranes (GE Healthcare). The membranes were incubated with the appropriate primary antibodies followed by the peroxidase-conjugated secondary antibody. The signals were quantified using the G: Box gel imaging system by Syngene (Frederick, Maryland).

### Immunoprecipitation

Immunoprecipitation was performed as described previously [19]. Briefly, about 200 µg of total cellular proteins were incubated with antibodies outlined in Table S1 overnight at 4 °C followed by adding 20 µL of protein A/G-agarose beads (Santa Cruz Biotechnology). The precipitates were washed four times with lysis buffer and boiled in SDS sample buffer. The supernatant was subjected to immunoblotting with appropriate antibodies.

### Lactate measurement

Serum was collected by centrifugation of clotted whole blood at 2500 rpm for 10 min. To measure lactate levels, 1 μL serum was diluted in 49 μL Lactate Assay Buffer (Sigma). Diluted serum samples (50 μL) were mixed with 50 μL reaction mix according to the manufacturer’s instruction (Sigma). The reaction was incubated for 30 min at room temperature and measured at 450 nm (A_450_). To measure intracellular lactate levels, cells were washed with cold PBS and homogenized in four volumes of the Lactate Assay Buffer (~150 μL) by pipetting up and down a few times. Insoluble material was removed by centrifugation at 14,000 rpm for 5 min. The supernatant was collected to measure lactate levels according to the manufacturer’s instruction. Intracellular lactate levels were normalized to total cell number.

### Immunofluorescence staining

Cells were washed once with PBS, fixed for 15 min in 4% paraformaldehyde at room temperature, permeabilized with 0.1% Triton X-100 for 20 min and blocked with 3% BSA (PBS) for 30 min. Cells were then incubated with primary antibodies (outlined in Table S1) diluted in 3% BSA (PBS) overnight at 4 °C. After three washes in PBS, fluorescein-labeled secondary antibodies diluted in PBS were added and incubated for 1 h at room temperature. After three washes, cells were mounted in Antifade Mounting Medium with DAPI (VecorLabs). Images were acquired using a CS SP8 Confocal Microscope (Leica).

### Chromatin immunoprecipitation (ChIP)

RAW 264.7 macrophages were treated with lactic acid (10 mM) or vehicle for 24 h. Cells were then washed twice with PBS and cross-linking was performed in 9 mL culture medium containing 1% formaldehyde at room temperature for 15 min. The reaction was stopped by the addition of glycine to the final concentration of 125 mM. Chromatin extraction was performed with the High Sensitivity ChIP Kit (Abcam) according to the manufacturer’s instructions. A total of 2 μg chromatin was used for the chromatin immunoprecipitation with anti-TEAD4 (Abcam) antibody overnight at 4 °C. Upon cross-link reversal and DNA purification, 1 μL of the eluted DNA was used for quantitative polymerase chain reaction (qPCR) with target region primers: 5′-GAAGTTTGCGCTCTCTCCCA-3′ and 5′-GATAGGGCGGGTCCTCAATC-3′.

### Permeability assay

HUVECs (2 × 10^5^) were cultured on transwell inserts (3.0 µm pore size) of a 24-well plate for two days (until confluent). Macrophage-derived exosomes (2.5 µg/mL) were added to the transwell inserts and incubated for 6 h. Then, 100 µL of PBS containing 1 µg/mL FITC-dextran was added to the upper chamber and 500 µL of PBS to the lower chamber. The 24-well plate with transwell inserts was incubated for 5 min and the concentration of FITC-dextran transferred to the lower chamber was determined using a Microplate Reader with excitation and emission wavelengths of 492 and 520 nm, respectively.

### Statistical analysis

Data were expressed as mean ± standard deviation (SD). A Student’s *t* test (two-sided) was used to compare two groups affected by one single variable. One-way ANOVA or two-way ANOVA with Turkey’s test was used to compare multiple data groups affected by one or two independent variables, respectively. All statistical analysis was carried out using SigmaPlot v11.0 software (Systat Software). Survival differences were determined using the Kaplan–Meier method and the Log-rank test. Differences were considered statistically significant at *P values* of *P*  < 0.05. The investigators were blinded to the group allocation during the experiment and data collection. Based on the power analysis, as well as the extensive experience with the mouse model of CLP sepsis, we estimated the number of mice per group that would be required to detect effects of interest at the *P*‘ < 0.05 level of significance. The numbers of technical replicates or biological replicates in each group are stated in the figure legends.

## Results

### Lactate and HMGB1 levels are increased in serum during polymicrobial sepsis

**Fig. 1:**
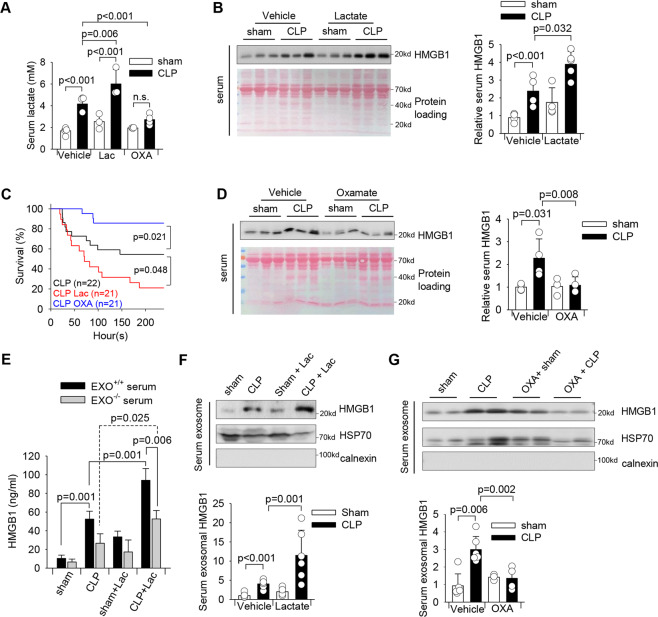
Elevated lactate levels contribute to increased circulating exosomal HMGB1 levels in polymicrobial sepsis. Lactate (0.5 g/kg body weight) was administrated through i.p. injection 6 h after CLP or sham surgery. To inhibit lactate production, sodium oxamate (OXA, 0.5 g/kg body weight) was i.p. injected 6 h before CLP or sham surgery. **A** Serum lactate levels were measured by Lactate Assay Kit (*n* = 5 for sham and CLP, *n* = 5 for sham + Lac, CLP + Lac, and sham + OXA, *n* = 4 for CLP + OXA, two-way ANOVA with Tukey’s test). **B** Serum HMGB1 levels among sham, CLP, sham + Lac, and CLP + Lac were assayed by western blot (*n* = 4 for sham, CLP and sham +Lac, *n* = 5 for CLP + Lac, two-way ANOVA with Tukey’s test). **C** The survival rate among CLP, CLP + Lac and CLP + OXA mice was compared by Kaplan–Meier test (*n* = 22 for CLP, *n* = 21 for CLP + Lac, and CLP + OXA). **D** Serum HMGB1 levels among sham, CLP, sham + OXA, and CLP + OXA were assayed by western blot (*n* = 3 for sham + OXA, *n* = 4 for sham, CLP, and CLP + OXA, two-way ANOVA with Tukey’s test). **E** HMGB1 levels in untouched serum and exosome-depleted serum of sham, CLP, Lac, and CLP + Lac were measured by ELISA (*n* = 4, two-way ANOVA with Tukey’s test). **F** Exosomes were isolated from the serum of sham, CLP, sham + Lac, and CLP + Lac mice. Exosome lysates were analyzed by western blot using antibodies against HMGB1, HSP70, and calnexin (*n* = 6 for each group, two-way ANOVA with Tukey’s test). **G** Exosomes were isolated from the serum of sham, CLP, OXA + sham, and OXA + CLP mice. Exosome lysates were analyzed by western blot using antibodies against HMGB1, HSP70 and calnexin (*n* = 3 for sham + OXA, *n* = 4 CLP + OXA, *n* = 6 for sham and CLP, two-way ANOVA with Tukey’s test). Values are mean ± SD. Lac lactic acid, OXA oxamate, CLP cecal ligation and puncture.

Clinical evidence has shown that the high serum lactate levels are associated with mobility and mortality of septic patients [20]. In addition, increased serum HMGB1 levels positively correlate with mortality in the late phase of sepsis [21]. Indeed, in a cecal ligation and puncture (CLP)-induced sepsis model, we observed significantly increased serum lactate (Fig. 1A) and HMGB1 (Fig. 1B) levels by 170 and 183% in CLP septic mice when compared with sham controls. To determine whether increased lactate levels could regulate the serum HMGB1 production during sepsis, we increased serum lactate levels by i.p. injection of lactate to mice 6 h after induction of sepsis and examined serum HMGB1 and lactate levels. As shown in Figs. 1A and B, lactate administration slightly increased serum lactate and HMGB1 levels in sham control mice. Remarkably, lactate administration to septic mice strongly increased serum lactate levels by 44% (Fig. 1A) and HMGB1 levels by 63% (Fig. 1B), while the decreased survival rate of septic mice (Fig. 1C), when compared with septic control mice. In contrast, suppressed lactate production by sodium oxamate, a specific LDH inhibitor, significantly attenuated CLP sepsis-increased serum levels of HMGB1 (Fig. 1D) and lactate (Fig. 1A), leading to improved survival outcome of septic mice (Fig. 1C). These data suggest that lactate plays an important role in sepsis-increased serum HMGB1 levels and mortality.

### Serum exosomes contain high levels of HMGB1 in polymicrobial sepsis

HMGB1 plays a critical role in multiple organ dysfunctions when released extracellularly in sepsis [8]. Exosomes have been demonstrated to mediate crosstalk between cells, tissues, and organs [22]. To examine whether HMGB1 could be carried by circulating exosomes during sepsis, we collected blood samples from sham control and septic mice treated with or without supplemental lactate and measured HMGB1 levels by ELISA in the serum with and without exosome depletion. Figure 1E shows that CLP sepsis markedly increased the serum levels of HMGB1 compared with sham control. Administration of supplemental lactate to septic mice further increased serum HMGB1 levels (Fig. 1E), which is consistent with the data shown in Fig. 1B. However, the serum HMGB1 levels in sham, CLP sepsis, Lac + sham, and Lac + CLP sepsis were significantly reduced by 36.8, 49.3, 48.2, and 44.0%, respectively after depletion of serum exosomes (Fig. 1E). The data suggest that circulating exosomes contain a significant amount of HMGB1.

Next, we focused on the role of exosomal HMGB1 during sepsis. We found that CLP sepsis markedly increased HMGB1 levels in serum exosomes compared with sham control (Fig. 1F). The size of isolated serum exosomes (125.02 ± 24.6) was measured by dynamic light scattering analysis (Figure S1A) and exosomes were further characterized for the presence of the exosomal markers and absence of endoplasmic reticulum (ER) protein calnexin (Figure S1B) [23, 24]. Importantly, elevating serum lactate levels by administration of lactate to septic mice further increased exosomal HMGB1 levels by 180% (Fig. 1F), when compared with septic control mice. In contrast, inhibition of lactate production by oxamate significantly suppressed sepsis-increased exosomal HMGB1 production (Fig. 1G). Together, these data demonstrate that sepsis increases serum exosomal HMGB1 levels and that lactate exerts a regulatory role in the exosome-mediated release of HMGB1.

### Lactate increases HMGB1 cytoplasmic accumulation and release via exosome secretion in macrophages

**Fig. 2:**
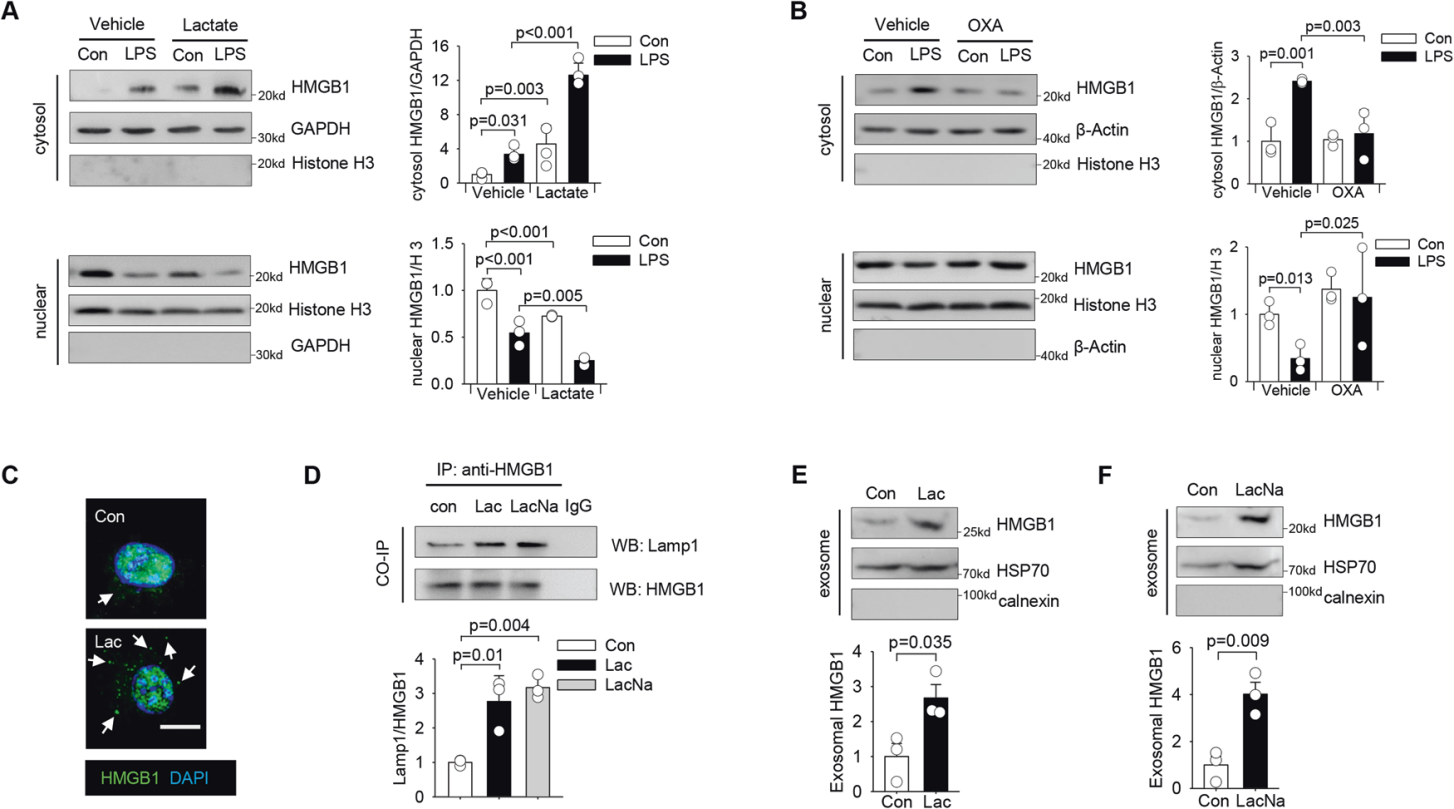
Lactate induces HMGB1 cytosol accumulation and release via exosome secretion in macrophages. **A** RAW 264.7 cells were pretreated with lactate (10 mM) for 30 min before LPS (500 ng/mL) stimulation for 6 h. Cytosol and nuclear HMGB1 levels were measured by western blot (*n* = 3, two-way ANOVA with Tukey’s test). **B** RAW 264.7 cells were pretreated with oxamate (20 mM) for 2 h before LPS (500 ng/ml) stimulation for 6 h. Cytosol and nuclear HMGB1 levels were measured by western blot (*n* = 3, two-way ANOVA with Tukey’s test). **C** Representative immunofluorescent staining images of RAW 264.7 cells treated with vehicle or lactate (10 mM) for 6 h show increased accumulation of HMGB1 in the cytoplasm (indicated by white arrows) of lactate-treated cells (Scale bar, 10 µm). **D** 200 µg of protein lysates were precipitated with anti-HMGB1 antibody followed by immunoblotting with anti-Lamp1 antibody shows lactate (lactic acid or sodium lactate) increased the interaction between HMGB1 and Lamp1 in RAW 264.7 cells (*n* = 3, *t* test). **E** and **F** RAW 264.7 cells were stimulated with lactic acid (E) or sodium lactate (F) for 24 h and macrophage-derived exosomes were isolated from the supernatant to examine the presence of HMGB1 protein by western blot (*n* = 3, *t* test). Values are mean ± SD. OXA sodium oxamate, Lac lactic acid, LacNa, sodium lactate.

Previous studies have shown that activated macrophages are one of the major sources of secreted HMGB1 during inflammation [11]. Consistently, we observed that LPS strongly promoted HMGB1 translocation from the nucleus to the cytoplasm in macrophages (Fig. 2A). Lactate administration further promoted HMGB1 cytoplasmic localization in LPS stimulated macrophages (Fig. 2A), while suppression of endogenous lactate production by oxamate attenuated LPS-induced HMGB1 cytoplasmic accumulation in macrophages (Fig. 2B). To further explore the regulatory role of lactate in HMGB1 secretion in macrophages, we then treated macrophages with lactate (10 mM) and observed that lactate alone can strongly induce accumulation of HMGB1 in the cytoplasm in macrophages (Fig. 2C). Flow cytometry analysis showed that lactate at concentrations up to 20 mM did not exhibit significant deleterious effects on macrophages (Figure S2), suggesting that lactate-increased HMGB1 cytoplasmic localization was not mediated by induction of cell death and apoptosis. A previous study has demonstrated that the cytosol HMGB1 is concentrated into secretory lysosomes for subsequent release [25]. Indeed, our immunoprecipitation data showed that lactate (lactic acid or sodium lactate) promoted co-localization of HMGB1 and Lamp1, a lysosomal marker (Fig. 2D). To further evaluate whether lactate could induce HMGB1 release from macrophages in exosomes, we isolated exosomes from supernatants of lactate-stimulated or control macrophages and examined exosomal HMGB1 protein levels. As shown in Figs. 2E and F, either lactic acid or sodium lactate significantly increased HMGB1 levels in the isolated exosomes. These data suggest that lactate can induce HMGB1 accumulation in the cytoplasm and promote its localization to the lysosomes for subsequent release via exosome secretion.

### Lactate directly promotes HMGB1 lactylation in macrophages

**Fig. 3:**
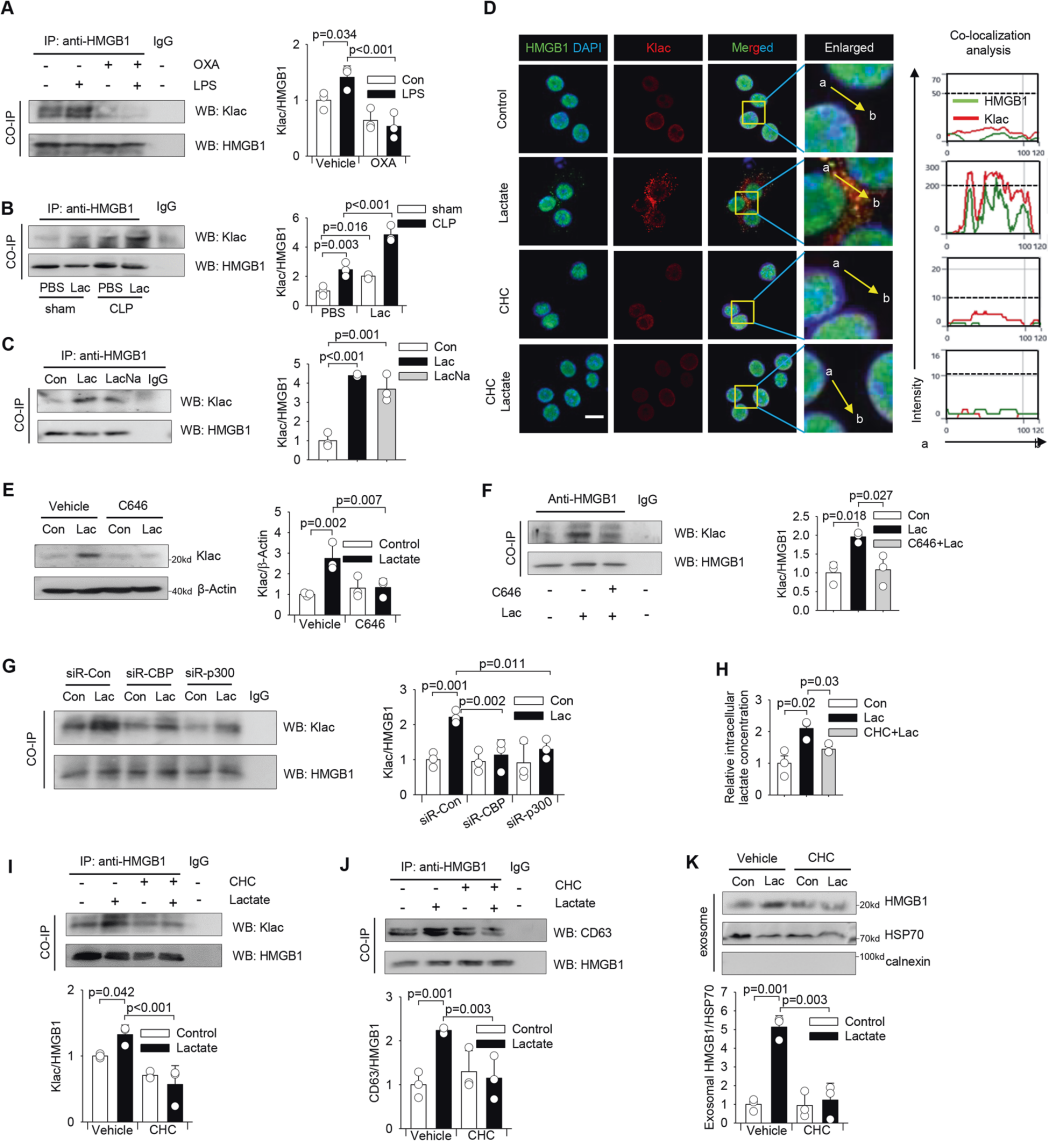
Lactate directly induces HMGB1 lactylation (Klac) in macrophages. **A** RAW 264.7 cells were pretreated with oxamate (20 mM) for 30 min followed by LPS (500 ng/mL) stimulation for 24 h. 200 µg of protein lysates were precipitated with anti-HMGB1 antibody followed by immunoblotting with anti-Klac antibody (*n* = 3, two-way ANOVA with Tukey’s test). **B** Sepsis was induced by CLP surgery followed by i.p. administration of lactate or vehicle. Peritoneal macrophages were prepared 24 h after CLP or sham surgery. Peritoneal macrophages in each group were isolated and cell lysates were precipitated with anti-HMGB1 antibody followed by immunoblotting with anti-Klac antibody (*n* = 3, two-way ANOVA with Tukey’s test). **C** 200 µg of protein lysates of lactic acid or sodium lactate-treated RAW 264.7 cells were precipitated with anti-HMGB1 antibody followed by immunoblotting with anti-Klac antibody showing lactate-induced Klac in HMGB1 immunocomplex (*n* = 3, two-way ANOVA with Tukey’s test). **D** RAW 264.7 cells were treated with CHC (3 mM) or DMSO for 2 h before lactate (10 mM) addition for another 24 h. HMGB1 (green) and Klac (red) co-localization was examined by confocal microscope (Scale bar, 10 µm). Nucleus was indicated by DAPI (blue) staining. Co-localization analysis was performed using Zeiss Zen microscope software. **E** and **F** RAW 264.7 cells were treated with C646 (5 µM) or vehicle for 2 h followed by lactate treatment for 24 h. Cell lysates were examined for Klac levels by western blot **D** or precipitated with anti-HMGB1 antibody and probed for Klac levels **E** (*n* = 3 for each group, two-way ANOVA with Tukey’s test). **G** CBP and p300 were silenced by transfection with specific siRNAs (40 nM) for overnight followed by lactate (10 mM) stimulation for 24 h. 200 µg of protein lysates were precipitated with anti-HMGB1 antibody followed by immunoblotting with anti-Klac antibody (*n* = 3, two-way ANOVA with Tukey’s test). **H** RAW 264.7 cells were pretreated with CHC (3 mM) or vehicle for 2 h followed by lactate (10 mM) addition for 24 h. Intracellular lactate levels were measured by Lactate Assay Kit. Not treated cells were used as control (*n* = 3 for each group, two-way ANOVA with Tukey’s test). **I**–**K** RAW 264.7 cells were treated with CHC (3 mM) or vehicle (DMSO) for 2 h followed by lactate treatment for 24 h. HMGB1 Klac levels were assayed by immunoprecipitation with anti-HMGB1 antibody followed by immunoblotting with anti-Klac antibody **I**. Interaction between HMGB1 and CD63 was assayed by immunoprecipitation with anti-HMGB1 antibody and probed for co-precipitation of CD63 in HMGB1 immunocomplex **J**. Exosomes were isolated from the supernatant and exosomal HMGB1 protein levels were examined by western blot **K** (*n* = 3 for each group, two-way ANOVA with Tukey’s test). Values are mean ± SD. Lac lactic acid, OXA sodium oxamate, LacNa sodium lactate, Klac lysine lactylation.

A recent study by Zhang showed that lactate directly modifies nuclear histones, a process of which has been identified as lysine lactylation (Klac) [14]. Interestingly, we found that LPS strongly elevated Klac levels in HMGB1 immunocomplex (Fig. 3A). To determine whether lactate is a mediator in LPS-induced HMGB1 lactylation, we suppressed endogenous lactate production by oxamate [26] prior to LPS stimulation and observed that oxamate strongly attenuated LPS-induced HMGB1 lactylation in macrophages (Fig. 3A). Consistently, we observed that isolated peritoneal macrophages from CLP septic mice had increased levels of lactylated HMGB1 (Fig. 3B). Administration of lactate to sham and CLP mice further increased HMGB1 lactylation levels in peritoneal macrophages (Fig. 3B). To further validate the effects of lactate on HMGB1 lactylation, we treated macrophages with lactate or hypoxic challenge and examined lactylation levels in the nuclear extracts. Our data show that either lactate treatment (Figure S3A) or increased lactate production by hypoxia challenge (Figure S3B) markedly increased Klac levels in the nucleus of macrophages. As expected, our immunoprecipitation data showed that lactate (lactic acid or sodium lactate) markedly increased Klac levels in the HMGB1 immunocomplex (Fig. 3C). Immunofluorescent staining also showed that lactate promoted the co-localization between Klac and HMGB1 in the cytoplasm of RAW 264.7 macrophages (Fig. 3D).

Lysine acetylase p300 has been reported to catalyze the transfer of the lactyl group from lactyl-CoA to histones in a cell-free system [14]. We investigated whether p300 and its homologue CREB-binding protein C (CBP) could also contribute to lactate-induced lysine lactylation in macrophages. To this end, we suppressed the activity of p300/CBP acetylases by C646 [27] and observed that p300/CBP inhibition significantly attenuated lactate-promoted Klac levels in macrophages (Fig. 3E). To further determine the role of p300/CBP activity in catalyzing HMGB1 lactylation, we performed immunoprecipitation with anti-HMGB1 antibody and examined Klac levels in the HMGB1 immunocomplex. Figure 3F shows that inhibiting enzyme activity of p300/CBP by C646 attenuated lactate-increased Klac levels in the HMGB1 immunocomplex. Similarly, silencing of either p300 or CBP by their specific siRNAs also attenuated lactate-induced HMGB1 lactylation in macrophages (Fig. 3G). Together, these data demonstrate that lactate can directly induce nuclear HMGB1 lactylation in a p300/CBP-dependent mechanism, which could be responsible for the translocation of HMGB1 from the nucleus to the cytoplasm.

### MCT facilitates lactate uptake into macrophages for HMGB1 lactylation

Next, we investigated whether macrophage could uptake extracellular lactate through monocarboxylic acid transporters (MCTs) for HMGB1 lactylation [28]. We blocked extracellular lactate uptake (Fig. 3H) in macrophages by MCT inhibitor (CHC) and observed that MCT inhibition suppressed lactate-induced increases in Klac levels in macrophages (Figure S3C). Importantly, MCT inhibition suppressed lactate-induced HMGB1 lactylation, as evidenced by decreased Klac levels in the HMGB1 immunocomplex (Fig. 3I). Immunofluorescent staining also showed that CHC dramatically reduced cytosol retention of lactylated-HMGB1 (Fig. 3D). In addition, CHC attenuated lactate-increased HMGB1 accumulation in lysosomes, as evidenced by decreased expression of lysosomal marker CD63 in the HMGB1 immunocomplex (Fig. 3J). Moreover, MCT inhibition reduced lactate-induced HMGB1 levels in macrophage-derived exosomes (Fig. 3K). Together, these data suggest that macrophages can uptake extracellular lactate through MCTs, which contributes to HMGB1 lactylation.

### Lactate promotes HMGB1 acetylation in macrophages

**Fig. 4:**
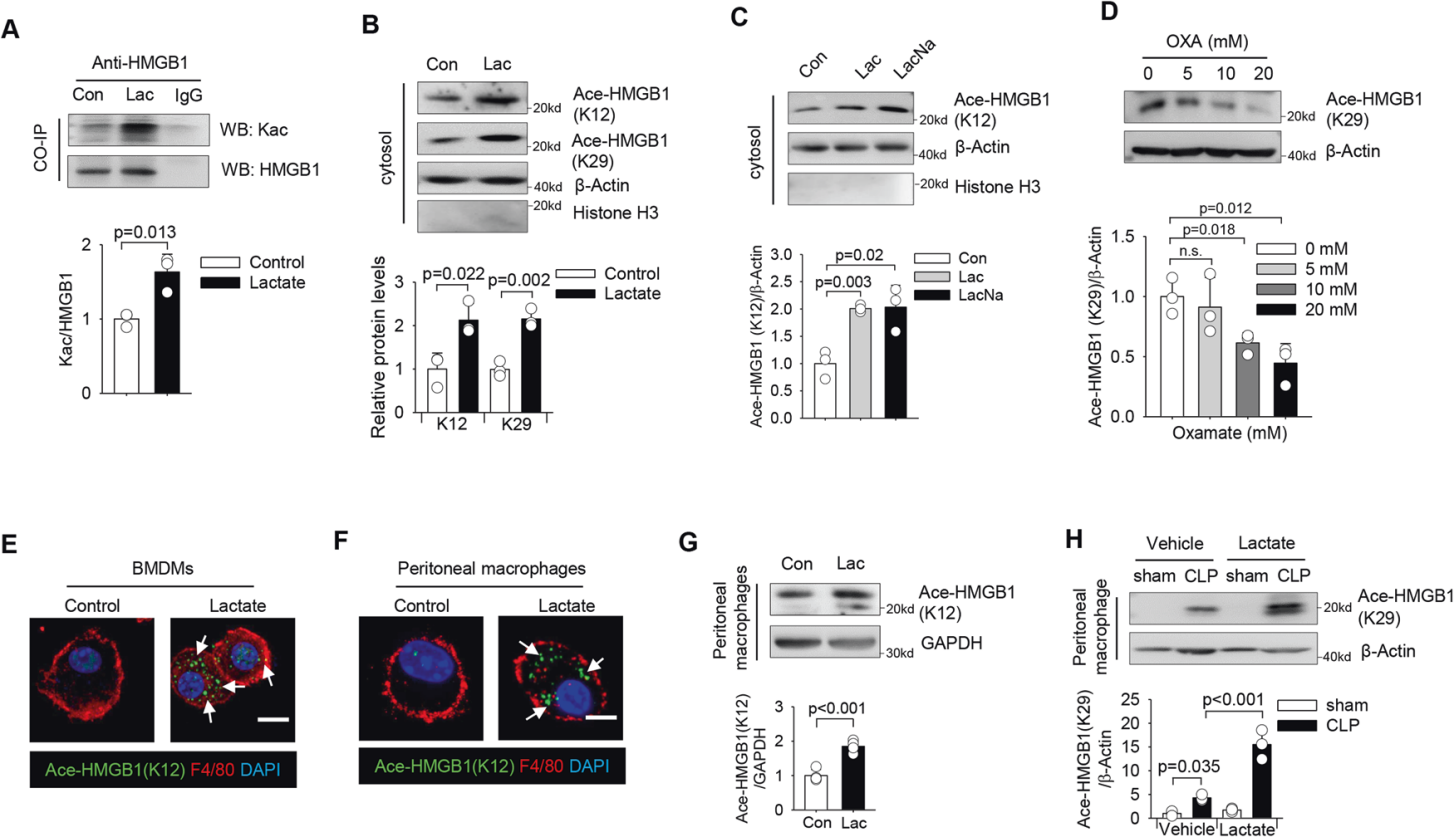
Lactate induces HMGB1 acetylation. **A** Cell lysates of lactate-treated RAW 264.7 macrophages were immunoprecipitated with anti-HMGB1 antibody and probed for acetylation (Kac) levels by western blot (*n* = 3 for each group, *t* test). **B** Western blot shows that lactate-induced HMGB1 acetylation at lysine 12 (K12) and 29 (K29) residues (*n* = 3 for each group, *t* test). **C** Both lactic acid and sodium lactate at 10 mM induced HMGB1 acetylation (*n* = 3 for each group, *t* test). **D** Oxamate treatment decreased HMGB1 acetylation levels in a dose-dependent manner in RAW 264.7 cells (*n* = 3 for each group, one-way ANOVA with Tukey’s test). **E–G** BMDMs and peritoneal macrophages were stimulated with lactate for 6 h and HMGB1 acetylation levels were assayed. Lactate-induced HMGB1 acetylation in BMDMs (**E**, indicated by white arrows). Immunofluorescent staining **F** and western blot analysis **G** show increased HMGB1 acetylation in lactate-treated peritoneal macrophages. **H** Sepsis was induced by CLP surgery followed by i.p. administration of lactate or vehicle. Peritoneal macrophages were isolated 24 h after CLP or sham surgery. Western blot was performed to detect HMGB1 acetylation levels in isolated peritoneal macrophages (*n* = 3, two-way ANOVA with Turkey’s test). Scale bar, 10 µm. Lac lactate, LacNa sodium lactate, OXA sodium oxamate.

To gain insights into whether lactate could also promote HMGB1 acetylation for its release, we examined lysine acetylation (Kac) levels in HMGB1 by immunoprecipitation following lactate treatment. As shown in Fig. 4A, lactate treatment markedly increased Kac in the HMGB1 immunocomplex, suggesting that lactate promotes HMGB1 lysine acetylation. Using specific antiactylated-HMGB1 antibodies, we observed increased acetylation levels of lysine 12 and 29 residues in HMGB1 upon lactate stimulation (Fig. 4B). To rule out the possibility that lactic acid induces HMGB1 acetylation by changing medium acidity, we also treated macrophages with sodium lactate and found that sodium lactate also increased acetylation levels in macrophages to a similar extend (Fig. 4C). Confocal microscopy also showed that lactate-increased levels of acetylated-HMGB1, which was mainly co-localized with Lamp1-positive organelles in the cytoplasm of macrophages (Figure S4). In contrast, inhibition of lactate production by oxamate (10 mM) significantly decreased HMGB1 acetylation in a dose-dependent manner (Fig. 4D). Similarly, we observed that lactate-promoted HMGB1 acetylation and cytoplasm distribution in both bone marrow-derived macrophages (BMDMs) (Fig. 4E) and peritoneal macrophages (Figs. 4F and 4G). Moreover, CLP-induced HMGB1 acetylation was further increased by lactate administration in peritoneal macrophages recovered from septic mice (Fig. 4H). Collectively, these data demonstrate that lactate stimulates HMGB1 acetylation and promotes HMGB1 translocation from the nucleus to the cytoplasm in macrophages.

### Lactate promotes HMGB1 acetylation by suppressing deacetylase SIRT1 activity

**Fig. 5:**
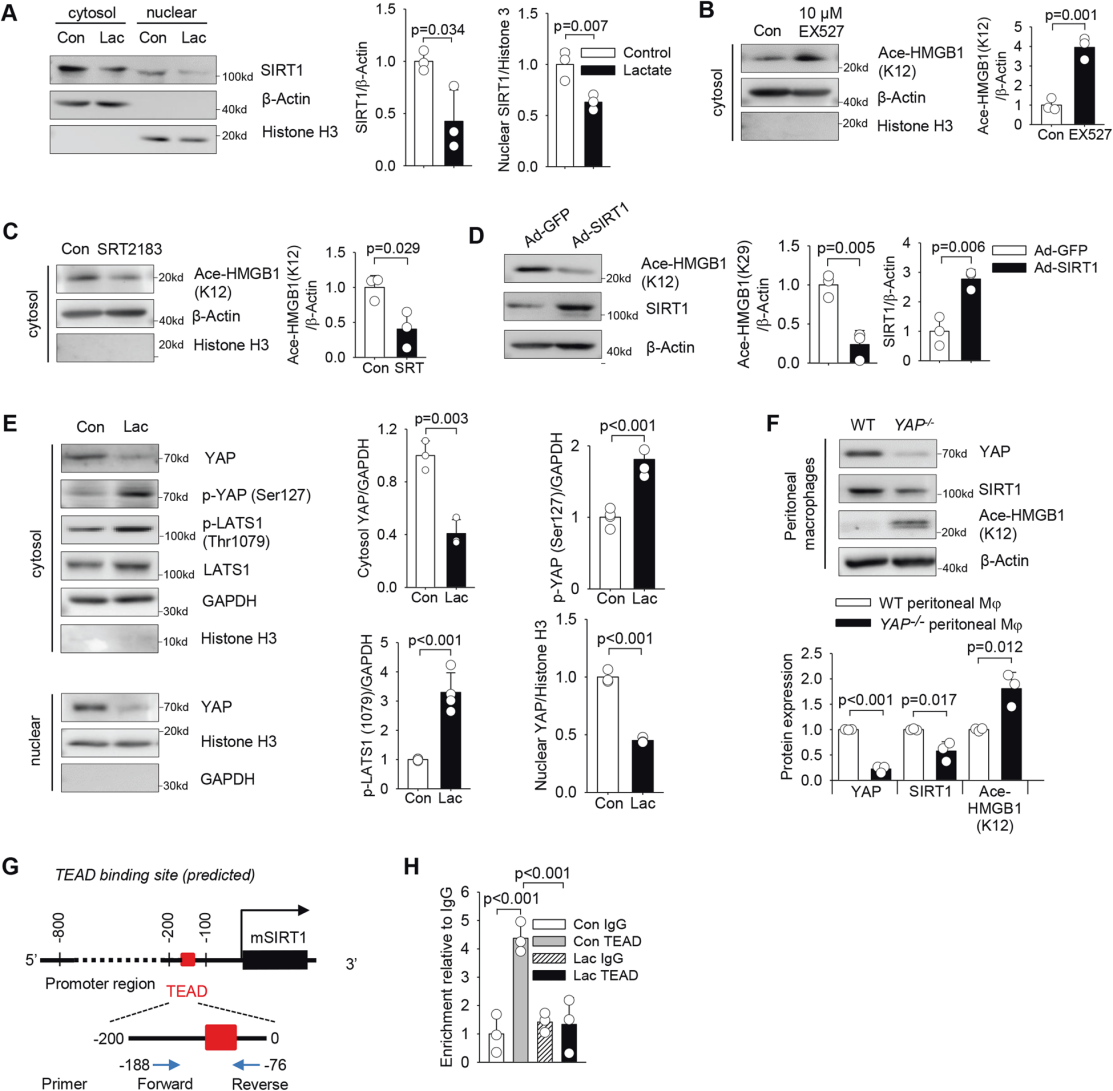
Lactate promotes HMGB1 acetylation through YAP-mediated suppression of deacetylase SIRT1 in macrophages. **A** Decreased expression of deacetylase SIRT1 in both cytosol and nuclear fractions of RAW 264.7 cells stimulated with lactate for 6 h (*n* = 3, *t* test). **B** Suppression of SIRT1 deacetylase activity by EX527 (10 μM) increased HMGB1 acetylation in 264.7 cells (*n* = 3, *t* test). **C** Activation of SIRT1 deacetylase by its activator SRT2183 (10 μM) decreased acetylated-HMGB1 expression in RAW 264.7 cells (*n* = 3, *t* test). **D** RAW 264.7 cells were transduced with Ad-GFP or Ad-SIRT1 overnight. Expression of SIRT1 and acetylated-HMGB1 (K12) were assessed by western blot (*n* = 3, *t* test). **E** RAW 264.7 cells were stimulated with lactate (10 mM) for 6 h and the cytosol expression of YAP, p-YAP (Ser127), p-LATS1 (Thr1079), LATS1, and nuclear expression of YAP were assayed by western blot (*n* = 3 for each group, *t* test). **F** Western blot analysis of YAP, SIRT1 and acetylated-HMGB1 (K12) expressions in wild type (WT) and YAP knockout (*YAP*^*−/−*^) peritoneal macrophages (*n* = 3, *t* test). **G** CiiiDER predicts a putative TEAD binding site locating on the promoter region of mouse *SIRT1*. **H** ChIP assay of the relative enrichment of TEAD4 on the promoter region of mouse *SIRT1* (*n* = 3, two-way ANOVA with Tukey’s test). Values are mean ± SD. Lac lactic acid.

To dissect the molecular mechanisms by which lactate promotes HMGB1 acetylation in macrophages, we re-analyzed the public gene-expression dataset (GSE115354) [14] obtained from Gene Expression Omnibus (GEO). We observed that class III lysine deacetylases expression was downregulated, while lysine acetylases expression was upregulated, in lactate-stimulated M1 BMDMs. SIRT1 is a NAD^+^-dependent class III protein deacetylase and has been reported to regulate the balance of HMGB1 acetylation/deacetylation [29]. Our data showed that lactate treatment markedly decreases the levels of cytosol and nuclear SIRT1 in macrophages (Fig. 5A), indicating that SIRT1 could participate in lactate-induced HMGB1 acetylation. Indeed, inhibition of SIRT1 deacetylase activity by selisistat (EX527), a specific SIRT1 deacetylase inhibitor, markedly increased HMGB1 acetylation (Fig. 5B). In contrast, both pharmacological activation of SIRT1 by its activator SRT2183 (Fig. 5C) and adenovirus-mediated SIRT1 overexpression (Fig. 5D) significantly decreased HMGB1 acetylation levels. These data suggest that lactate suppresses the expression of SIRT1 deacetylase, which tilts the balance of HMGB1 acetylation/deacetylation toward acetylation in macrophages.

### Lactate-suppressed SIRT1 expression is mediated by YAP inactivation

YAP has been reported to preserve SIRT1 expression and activation [30]. As a key component of Hippo signaling, LATS activation directly increases YAP phosphorylation, resulting in YAP degradation [19]. Consistently, we found that lactate treatment markedly increased the protein levels of phosphorylated-LATS1 and phosphorylated-YAP, while decreased cytosolic and nuclear YAP protein expression in RAW 264.7 macrophages (Fig. 5E). Confocal microscope examination revealed the co-localization between YAP and SIRT1 in the nucleus of control macrophages, which was decreased by lactate treatment (Figure S5). These data suggest that lactate can induce YAP phosphorylation and degradation mediated by LATS1 activation, thereby suppressing SIRT1 nuclear translocation with YAP.

To explore the role of YAP in the regulation of SIRT1 expression and HMGB1 acetylation following lactate treatment, we isolated peritoneal macrophages from both wild type (WT) and macrophage-specific YAP knockout (*YAP*^*fl/fl*^*; Lyz2-Cre*^*+*^) mice (Figure S6A) and examined SIRT1 expression and HMGB1 acetylation. The purity of isolated peritoneal macrophages was assessed by flow cytometry (Figure S6B). YAP knockout was confirmed by immunofluorescent staining (Figure S6C) and western blotting (Fig. 5F). We found that YAP deficiency significantly decreased SIRT1 levels and increased HMGB1 acetylation in peritoneal macrophages (Fig. 5F). In contrast, forced YAP expression by transducing macrophages with adenovirus expressing YAP (Ad-YAP) markedly increased SIRT1 nuclear levels and suppressed HMGB1 acetylation (Figure S7), suggesting that YAP is needed for maintaining the SIRT1 expression and nuclear translocation for the homeostasis of HMGB1 acetylation/deacetylation in macrophages.

Next, we inhibited YAP transcriptional activity by verteporfin (VP) [31] and examined the expression of SIRT1 and HMGB1 acetylation levels. As expected, VP suppressed both mRNA (Figure S8A) and protein (Figure S8B) levels of SIRT1, while increased HMGB1 acetylation levels (Figure S8B) in a dose-dependent manner, indicating that YAP plays a role in controlling the transcription of SIRT1. Utilizing a transcription factor binding site (TFBS) analysis tool [32], we identified a potential binding site for TEAD on the SIRT1 promoter region (Fig. 5G). We then performed a chromatin immunoprecipitation (ChIP) assay and examined the enrichment of TEAD on the SIRT1 promoter region. As shown in Fig. 5H, there was a significant enrichment of TEAD on SIRT1 promoter in macrophages. However, lactate treatment markedly reduced the binding of TEAD to the SIRT1 promoter (Fig. 5H), which was correlated with lactate-suppressed YAP nuclear expression (Fig. 5E). Together, these data demonstrate that YAP activation and nuclear translocation are essential for SIRT1 nuclear translocation to maintain homeostasis of HMGB1 acetylation/deacetylation in macrophages. Moreover, lactate-induced YAP phosphorylation and degradation suppress SIRT1 transcription, thereby promoting HMGB1 acetylation.

### Lactate-activated β-arrestin2 recruits p300/CBP for HMGB1 acetylation in macrophages

**Fig. 6:**
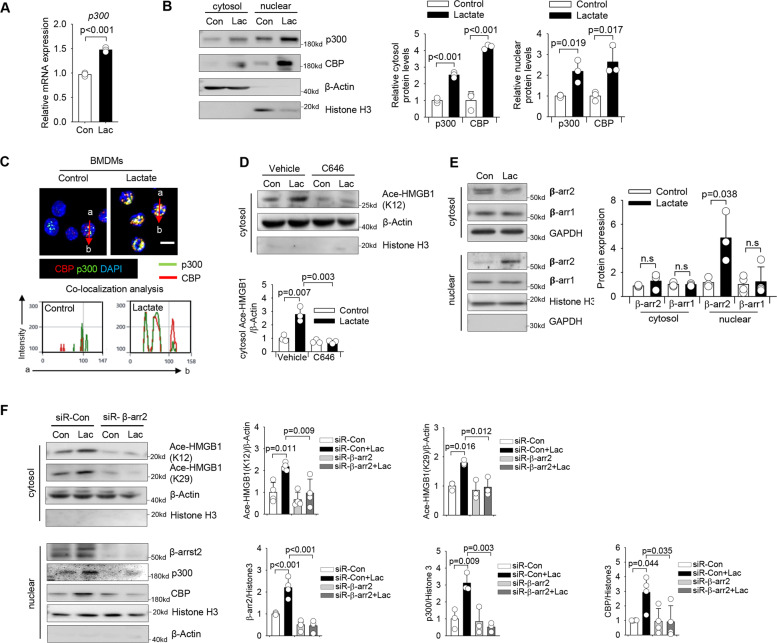
Lactate promotes HMGB1 acetylation via β-arrestin2-dependent recruitment of acetylases p300/CBP in macrophages. **A** Lactate upregulated p300 mRNA level in RAW 264.7 cells (*n* = 3, *t* test). **B** Western blot shows that lactate increased the expression of protein CBP and p300 in RAW 264.7 cells (*n* = 3 for each group, *t* test). **C** Representative images of expression and localization of CBP (red) and p300 (green) in lactate-treated RAW 264. 7 cells. The nucleus was stained with DAPI (blue). Co-localization analysis was performed using Zeiss Zen microscope software. (Scale bar, 10 µm). **D** Inhibited activity of acetylases p300/CBP by C646 suppressed lactate-induced HMGB1 acetylation (*n* = 3, two-way ANOVA with Turkey’s test). **E** Western blot shows that lactate-increased β-arrestin2 (β-arr2), but not β-arrestin1 (β-arr1), nuclear expression in the nucleus of RAW 264.7 cells (*n* = 3 for each group, *t* test). **F** RAW 264.7 cells were transfected with control siRNA (siR-Con) or β-arrestin2 specific siRNA (siR-β-arr2) overnight before lactate treatment. Expression of cytosol acetylated-HMGB1 (k12) and acetylated-HMGB1 (K29), and expression of nuclear β-arrestin2, p300 and CBP were examined by western blot (*n* = 4 for K12, *n* = 3 for K29, *t* test). Values are mean ± SD. Lac lactic acid, β-arr β-arrestin.

We next investigated the expression of lysine acetylases upon lactate administration and observed that lactate treatment markedly increased the transcription level of p300 (Fig. 6A) and increased cytosol and nuclear protein levels of p300 and CBP (Fig. 6B). Confocal microscopy (Fig. 6C) and immunoprecipitation (Figure S9A and S9B) also showed a significantly increased interaction between p300 and CBP on lactate treatment. Notably, we observed that lactate increased the levels of p300 and CBP in the HMGB1 immunocomplex (Figure S9C). To determine whether acetylase activity of p300/CBP is required for lactate-induced HMGB1 acetylation, macrophages were treated with C646 before lactate administration. As shown in Fig. 6D, inhibition of p300/CBP acetylase activity significantly attenuated lactate-induced increases in the acetylated-HMGB1 levels in the cytosol. Similarly, silencing of either p300 (Figure S10A) or CBP (Figure S10B) by siRNA transfection partially blocked lactate-induced HMGB1 acetylation in macrophages, suggesting that acetylases p300/CBP are involved in lactate-promoted HMGB1 acetylation.

β-arrestins have been reported to translocate into the nucleus to regulate gene transcription by enhancing histone acetylation in a p300-dependent manner [33]. Interestingly, we observed that lactate specifically increased the nuclear expression of β-arresin2 but not β-arresin1 (Fig. 6E) in macrophages. Immunofluorescent staining further showed increased nuclear translocation of β-arrestin2 and cytosol accumulation of acetylated-HMGB1 in lactate-treated macrophages (Figure S11A). Similarly, treatment of BMDMs with lactate also increased nuclear β-arresin2 levels (Figure S11B) and cytosol levels of acetylated-HMGB1 (Fig. 4E). However, silencing of β-arresin2 by siRNA transfection before lactate treatment markedly attenuated lactate-induced nuclear accumulation of p300/CBP and HMGB1 acetylation (Fig. 6F). Collectively, our data demonstrate that β-arristin2-dependent recruitment of p300/CBP into the nucleus plays an important role in lactate mediated HMGB1 acetylation.

### Lactate-induced HMGB1 acetylation is mediated by GPR81 signaling

**Fig. 7:**
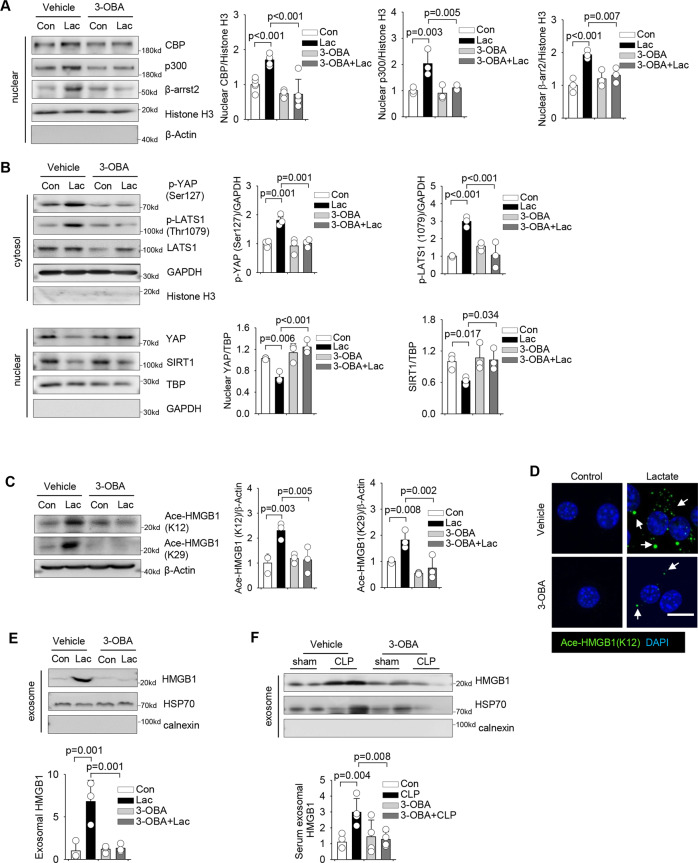
Lactate-induced HMGB1 acetylation is mediated by GPR81 signaling. **A**–**D** RAW 264.7 cells were treated with 3-OBA (5 mM) for 2 h followed by lactate administration for 6 h. Blockage of GPR81 by 3-OBA attenuated lactate-increased nuclear expression of CBP (*n* = 5), p300 (*n* = 3) and β-arrestin2 (*n* = 3) **A**. Blockage of GPR81 by 3-OBA attenuated lactate-induced phosphorylation of YAP (*n* = 3) and LATS1 (*n* = 3), and lactate-suppressed nuclear YAP expression (*n* = 3) and SIRT1 expression (*n* = 3) **B**. Lactate-promoted acetylation of HMGB1 was attenuated by 3-OBA pretreatment **C**. Immunofluorescent staining shows that blockage of GPR81 by 3-OBA diminished lactate-induced HMGB1 acetylation (green) and its cytoplasmic accumulation (indicated by white arrows) **D**. **E** RAW 264.7 cells were pretreated with 3-OBA (5 mM) for 2 h followed by lactate treatment for 24 h. Exosomes were isolated from the culture medium and exosomal HMGB1 protein levels were examined by western blot. (*n* = 3) **F** 3-OBA (0.5 g/kg body weight) was i.p. injected 6 h prior to CLP or sham surgery. Serum was collected 24 h following CLP for exosome isolation. Protein levels of HMGB1 in serum exosomes of sham, CLP, 3-OBA + sham and 3-OBA + CLP mice were examined by western blot (*n* = 4). Values are mean ± SD. Two-way ANOVA with Turkey’s test was performed. Scale bar, 10 µm. Lac lactate.

G protein-coupled receptor 81 (GPR81) is reported to be a lactate receptor in mediating intracellular signaling [34]. To investigate the role of GPR81 in lactate-induced HMGB1 acetylation, we treated macrophages with the GPR81 antagonist 3-hydroxy-butyrate acid (3-OBA) 2 h prior to lactate treatment. Figure 7A shows that GPR81 inhibition attenuated lactate-induced nuclear accumulation of β-arrestin2, p300, and CBP in macrophages. GPR81 inhibition also prevented lactate-induced phosphorylation of LATS1 and YAP, and preserved YAP expression in macrophages (Fig. 7B). Importantly, blockage of GPR81 signaling significantly attenuated lactate-promoted HMGB1 acetylation (Figs. 7C and 7D) and suppressed lactate-increased exosomal HMGB1 levels (Fig. 7E), when compared with control groups. To further confirm whether in vivo inhibition of GPR81 signaling will attenuate sepsis-increased serum exosomal HMGB1 levels, mice were administrated with 3-OBA by i.p. injection before CLP/sham surgery and HMGB1 expression in serum exosomes was analyzed. As shown in Fig. 7F, GPR81 inhibition significantly decreased HMGB1 levels in serum exosomes of septic mice. Together, these data provide evidence that GPR81 plays an important role in lactate-promoted HMGB1 acetylation through β-arrestin2-mediated recruitment of p300/CBP acetylases and YAP-dependent suppression of SIRT1 deacetylase in the nucleus.

### Macrophage-derived exosomal HMGB1 induces endothelial dysfunction

**Fig. 8:**
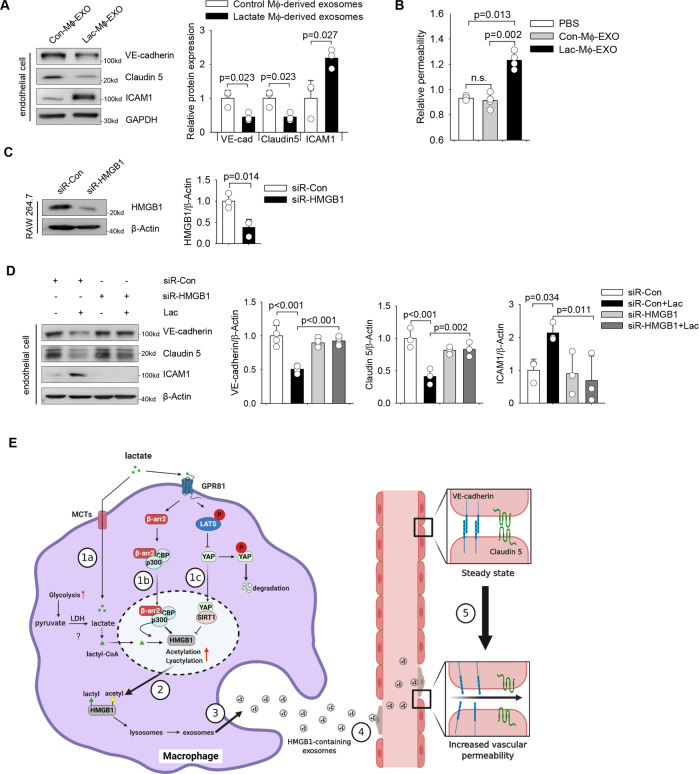
Macrophage-derived exosomal HMGB1 induces endothelial dysfunction. **A** RAW 264.7 cells were stimulated with lactate (10 mM) for 24 h and exosomes were isolated from the supernatant. HUVECs were incubated with 2.5 µg/mL exosomes, derived from either control macrophages or lactate-treated macrophages, for 6 h. Western blot shows that exosomes derived from lactate-treated macrophages reduced the expression of VE-cadherin and Claudin5, while increased ICAM1 expression, in HUVECs, as compared with HUVECs incubated with exosomes derived from control cells (*n* = 3 for each group, *t* test). **B** HUVECs were seeded into the upper chambers of a transwell system. HUVECs monolayers were then treated with 2.5 µg/mL exosomes, derived from either control macrophages or lactate-treated macrophages, for 6 h followed by addition of FITC-dextran. Fluorescence was quantified in the lower chamber 5 min after administration of FITC-dextran. Exosomes derived from lactate-treated macrophages increased the endothelial permeability (*n* = 4 for each group, *t* test). **C** RAW 264.7 cells were transfected with HMGB1 siRNA or control siRNA for 24 h. HMGB1 expression was examined by western blot (*n* = 3, *t* test). **D** RAW 264.7 cells were transfected with HMGB1 siRNA or control siRNA for 24 h followed by lactate stimulation. Exosomes were collected from supernatants and added to endothelial cell culture. Expression of endothelial VE-cadherin, Claudin 5, and ICAM1 was assayed by western blot (*n* = 4 for VE-cadherin, *n* = 3 for claudin 5 and ICAM1, Two-way ANOVA with Turkey’s test). **E** Scheme of lactate-induced HMGB1 lactylation/acetylation and exosomal release during sepsis. During sepsis, macrophages can uptake lactate through monocarboxylate transporters (MCTs), which leads to HMGB1 lactylation in a p300/CBP-dependent mechanism (1a). In addition, extracellular lactate increases β-arrestin2-recruited acetylase p300/CBP nuclear translocation (1b) and suppresses LATS/YAP-mediated deactivation of deacetylation SIRT1 (1c), via GPR81-dependent signaling, resulting in increased acetylation of HMGB1. Lactylated/acetylated HMGB1 in turn, is translocated into lysosomes in the cytoplasm of macrophages (2) and released via exosome secretion (3) from macrophages. Secreted exosomal HMGB1 further disrupts endothelium barrier function (4) by decreasing VE-cadherin and claudin 5 expressions and increasing ICAM1 expression in endothelial cells. Values are mean ± SD. EXO exosome, Mɸ macrophage, Lac lactate.

Increased circulating HMGB1 levels contribute to endothelial injuries [35]. However, the role of exosomal HMGB1 in sepsis pathogenesis is unclear. We observed that exosomes derived from lactate-treated macrophages significantly decreased VE-cadherin and Claudin 5, while increased ICAM1, protein levels in HUVECs (Fig. 8A).

In addition, exosomes from lactate-treated macrophages significantly increased permeability of endothelium, while exosomes from control macrophages have no such effect (Fig. 8B). In contrast, silencing of HMGB1 by its specific siRNA in macrophages before exosome enrichment (Fig. 8C) protected against exosomal HMGB1-decreased expression of VE-cadherin and Claudin 5, and exosomal HMGB1-induced ICAM1 expression in endothelial cells (Fig. 8D). We have shown that lactate-induced HMGB1 release was mediated by downregulation of YAP expression and activation of p300/CBP acetylases. Next, we silenced either p300 or CBP expression by specific siRNAs and overexpressed YAP by adenovirus transduction in RAW 264.7 cells before lactate stimulation. As shown in Figure S12, downregulation of p300 (A) and CBP (B), as well as upregulation of YAP (C), attenuated exosomal HMGB1-decreased expression of VE-cadherin and Claudin5, and exosomal HMGB1-induced ICAM1 expression in endothelial cells. Collectively, these data suggest that lactate-promoted HMGB1 lactylation, acetylation, and release via exosome secretion. HMGB1-containing exosomes further disrupts endothelial adherens and tight junction proteins and increases adhesion molecule expression, leading to the endothelial barrier dysfunction (Fig. 8E).

## Discussion

The present study demonstrated a novel role of lactate in promoting HMGB1 lactylation/acetylation and release via exosome secretion in macrophages. As an important damage-associated molecular pattern (DAMP) molecule, circulating HMGB1 plays a critical role in progression and late mortality of sepsis [10, 21]. Post-translational acetylation of lysine residues in HMGB1 underlies the major mechanism for HMGB1 release from activated macrophages during inflammation [11, 36, 37]. Bonaldi and colleagues previously reported that HMGB1 acetylation is regulated by histone acetylases, such as CBP, p300, and PCAF, indicating that HMGB1 acetylation may be part of a general acetylation wave in activated macrophages [11]. Consistently, we have found that lactate stimulates p300/CBP nuclear translocation and interaction with HMGB1, thus promoting HMGB1 acetylation. Moreover, stimulation of G protein-coupled receptor induced an association of β-arrestins and p300 in the nucleus, resulting in histone acetylation and increased transcription of specific genes [33]. GPR81 is a family member of G protein-coupled receptors (GPCRs) and has been identified as a lactate receptor [34]. Here we showed that the addition of lactate to macrophages induces GPR81-mediated activation of β-arrestin2, which contributes to nuclear accumulation of p300/CBP for HMGB1 acetylation. In addition to increased p300/CBP acetylase activity, we also discovered that lactate could strongly suppress the gene expression of SIRT1, an NAD^+^-dependent deacetylase that controls the acetylation/deacetylation balance of histones and nonhistone substrates in the nucleus [38, 39]. Interestingly, Yan et al. reported that YAP could preserve SIRT1 expression and function in gastric cancer [30]. Of note, our recent publication showed that lactate inhibits YAP activity via GPR81-dependent signaling in LPS-treated macrophages [40]. In this study, we further demonstrated that YAP is required for SIRT1 transcription and nuclear retention through direct interaction with SIRT1. Lactate induces YAP phosphorylation and degradation by activating LATS, thereby decreasing SIRT1 nuclear translocation, and ultimately causes enhanced HMGB1 acetylation.

A groundbreaking discovery by Zhang et al. showed that lactate is an epigenetic regulator for the modulation of histones by introducing a lactyl functional group to histones and thus modulating specific gene transcription [14]. This newly discovered process whereby lactate mediates histone lysine modification is referred as lactylation (Klac). Notably, we unraveled that lactate could induce HMGB1 lactylation and promote HMGB1 release from macrophages. Indeed, either suppressed endogenous lactate production by glycolysis inhibition or blockage of extracellular lactate uptake could attenuate HMGB1 lactylation in macrophages. It is suggested that the rate of lactylation is largely dependent on the lactyl-CoA concentration and the acetylase enzymes that can catalyze the transfer of the lactyl group from lactyl-CoA to histones [14]. We demonstrated that p300/CBP acetylases are important writers for HMGB1 lactylation in macrophages. However, there are several limitations in the current study. Although it is suggested that lactylation of histones has a distinct role in regulating gene transcription as compared with histone acetylation [14], it remains to be investigated whether lactylation can regulate or compete with acetylation in mediating HMGB1 cytoplasmic localization and subsequent release. In addition, future studies utilizing mass spectrometry and site mutation assessment to reveal the specific lactylation sites in HMGB1 will be needed. Moreover, while we found that lactate-induced extracellular release of HMGB1 in exosomes caused endothelial cell injury, the mechanism is not known. It is possible that exosomal HMGB1 is transported into endothelial cells by exosome internalization [41], instead of directly binding to intracellular receptors, such as RAGE and TLRs [42]. Indeed, a recent study by Lan et al. showed that internalized HMGB1 activates serine protease enzyme cathepsin [43], which could result in the disrupted endothelial cell-cell junctions [44].

In summary, the present study demonstrates a novel role of lactate in HMGB1 lactylation/acetylation in macrophages. Lactate is directly involved in HMGB1 lactylation by introducing lactyl groups to the lysine residues of HMGB1, which depends on p300 acetylase activity. Moreover, lactate-triggered HMGB1 acetylation can be attributed to two important mechanisms: i) increasing β-arrestin2-mediated activation of p300/CBP acetylase, and ii) suppressing YAP activation and SIRT1 deacetylase activity, which leads to HMGB1 acetylation in macrophages. The lactylated/acetylated HMGB1 is then translocated from the nucleus to the cytoplasm and subsequently released into circulation via exosome secretion. Secreted exosomal HMGB1 further disrupted endothelium integrity and increased vascular permeability. In conclusion, the present study provides a novel mechanistic basis for the deleterious effects of lactate during sepsis through promoting HMGB1 release. Our data suggest that glycolysis-derived lactate and lactate-associated signaling could be potential targets for suppressing HMGB1 release from macrophages and improving survival outcome in polymicrobial sepsis.

## Supplementary information

Supplemental Figure 1

Supplemental Figure 2

Supplemental Figure 3

Supplemental Figure 4

Supplemental Figure 5

Supplemental Figure 6

Supplemental Figure 7

Supplemental Figure 8

Supplemental Figure 9

Supplemental Figure 10

Supplemental Figure 11

Supplemental Figure 12

Supplemental Table 1

Supplementary Figure legends

## Data Availability

All data needed to evaluate the conclusions in the paper are present in the paper and/or the Supplementary Materials. Additional data related to this paper may be requested from the authors.
