# Supplementary figures and images for: Lactate promotes macrophage HMGB1 lactylation, acetylation, and exosomal release in polymicrobial sepsis
Source: Cell Death Differ. 2021 Aug 6;29(1):133–46. doi: 10.1038/s41418-021-00841-9 (PMC8738735; doi:10.1038/s41418-021-00841-9)

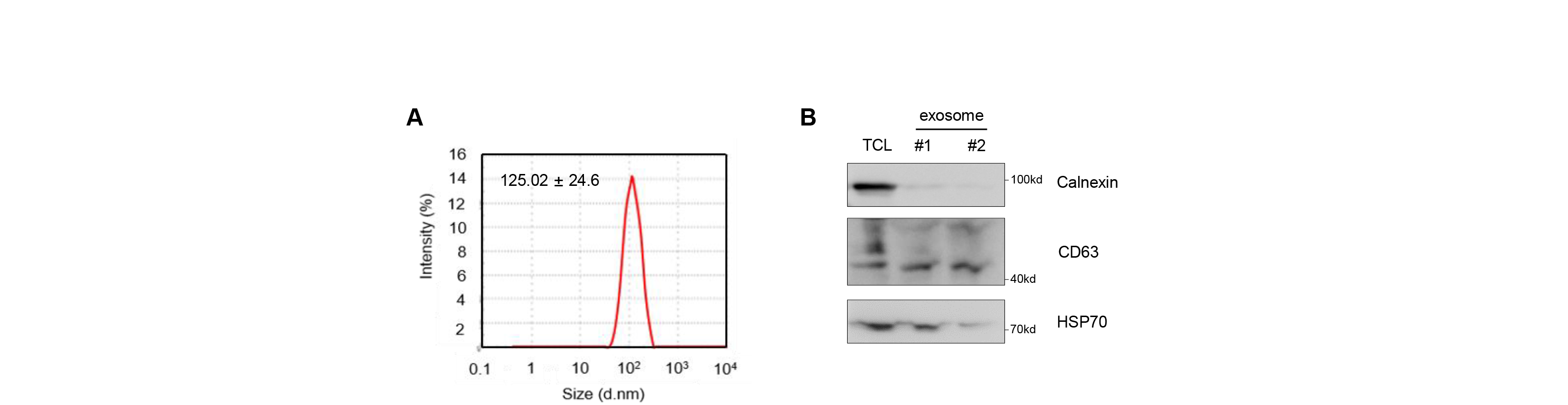

Supplement: Supplementary file 1 — Supplemental Figure 1 [file 41418_2021_841_MOESM1_ESM.tif]

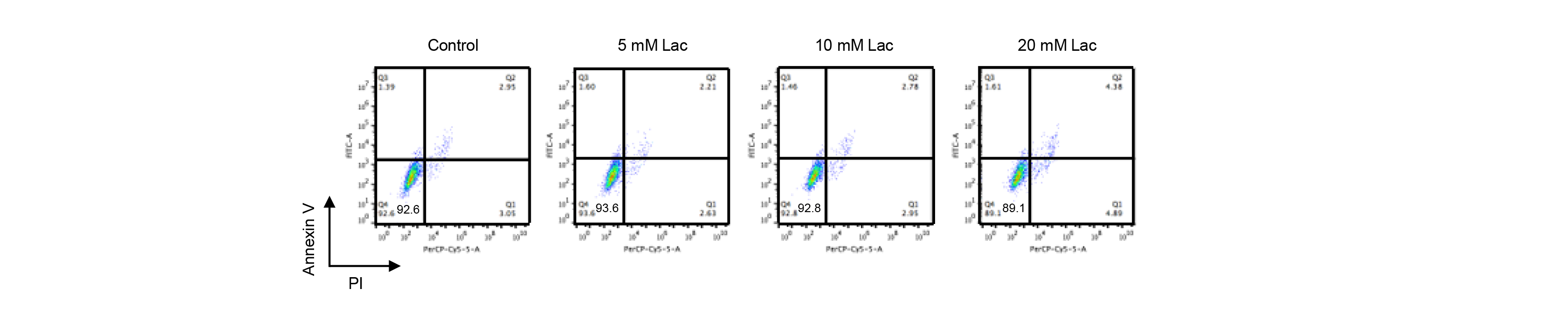

Supplement: Supplementary file 2 — Supplemental Figure 2 [file 41418_2021_841_MOESM2_ESM.tif]

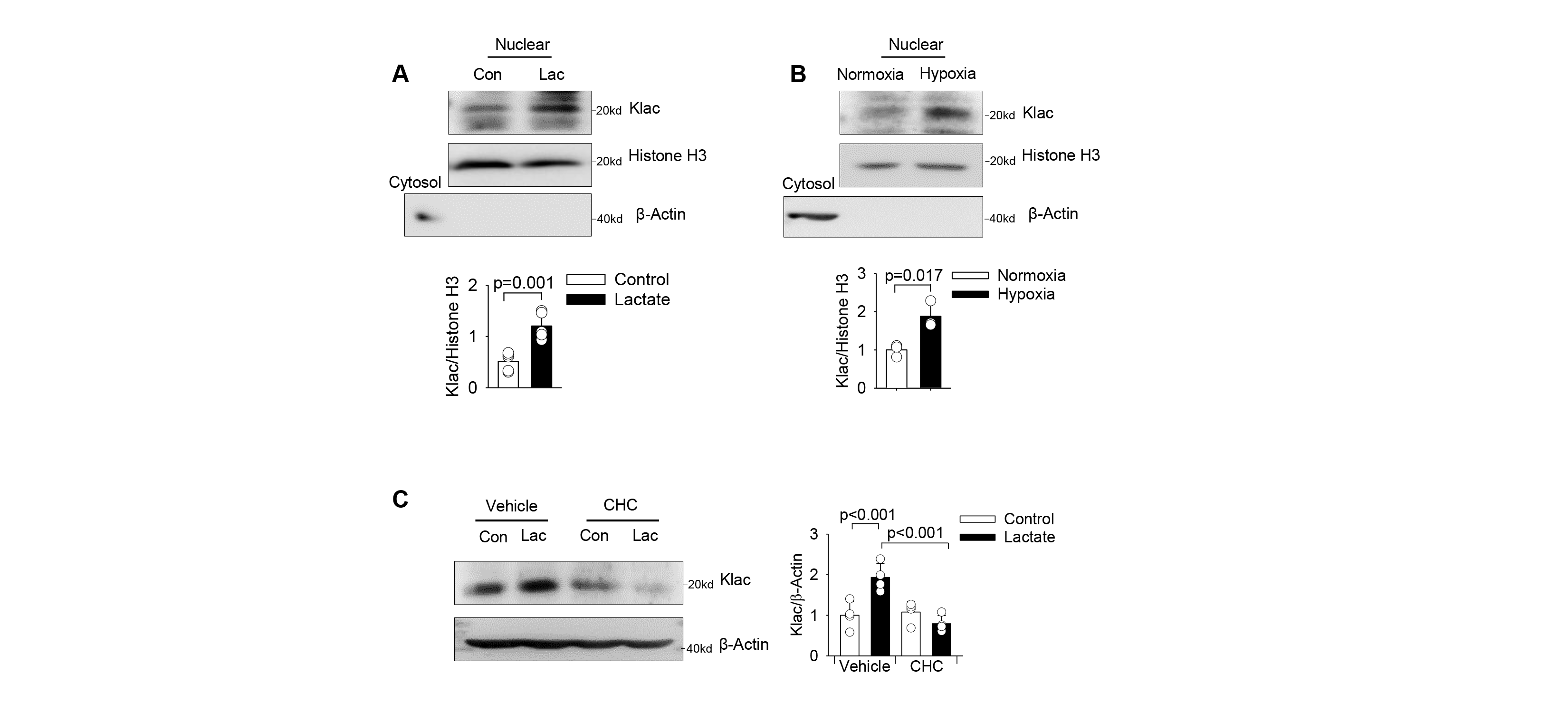

Supplement: Supplementary file 3 — Supplemental Figure 3 [file 41418_2021_841_MOESM3_ESM.tif]

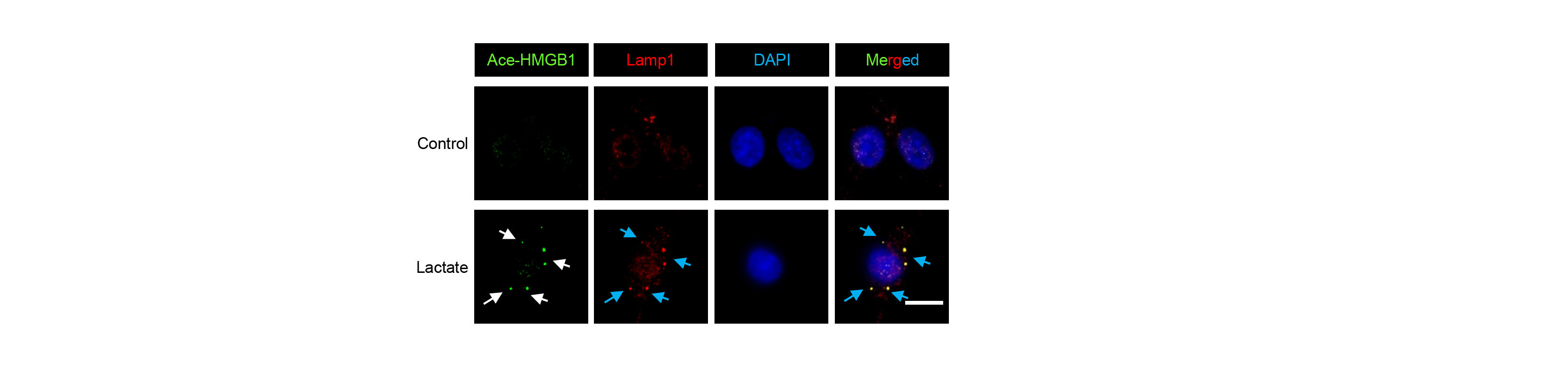

Supplement: Supplementary file 4 — Supplemental Figure 4 [file 41418_2021_841_MOESM4_ESM.tif]

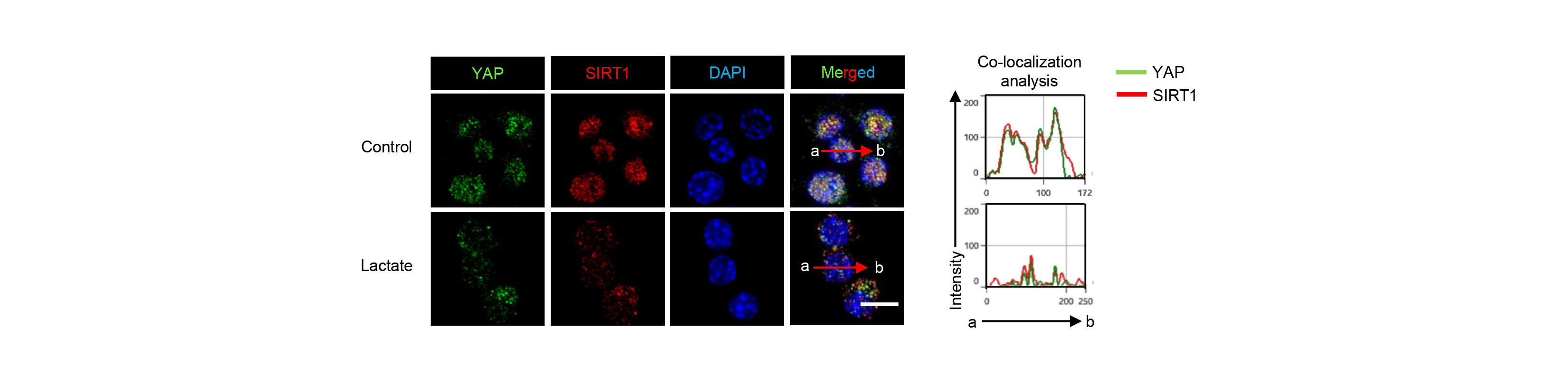

Supplement: Supplementary file 5 — Supplemental Figure 5 [file 41418_2021_841_MOESM5_ESM.tif]

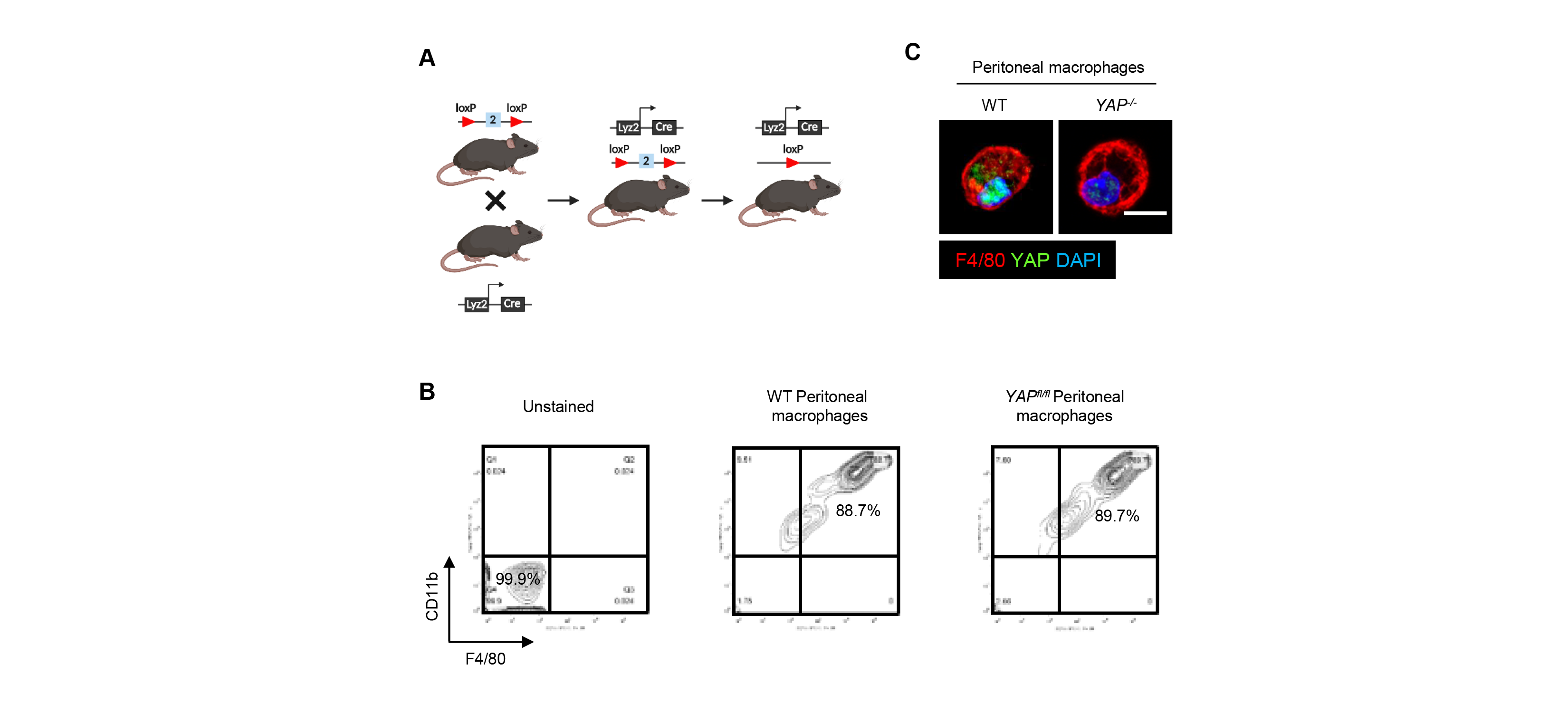

Supplement: Supplementary file 6 — Supplemental Figure 6 [file 41418_2021_841_MOESM6_ESM.tif]

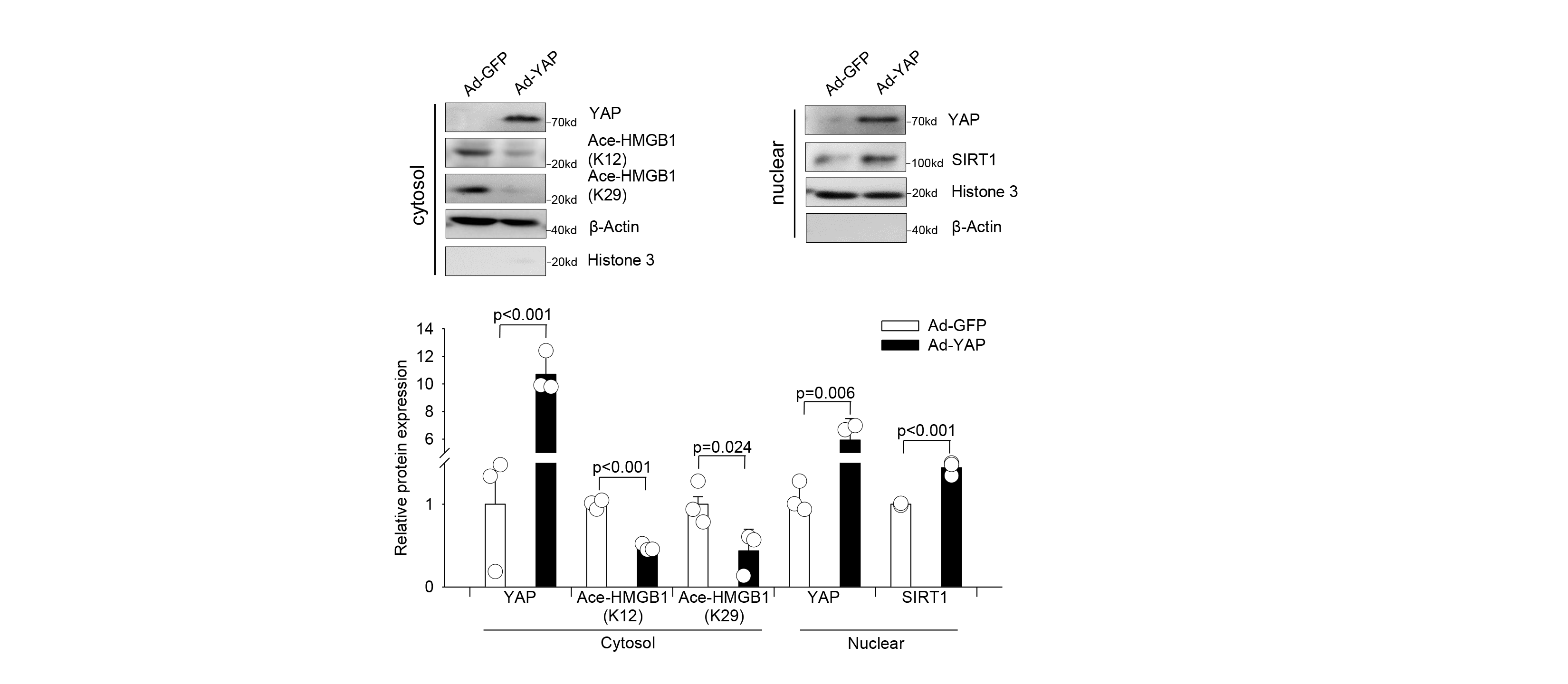

Supplement: Supplementary file 7 — Supplemental Figure 7 [file 41418_2021_841_MOESM7_ESM.tif]

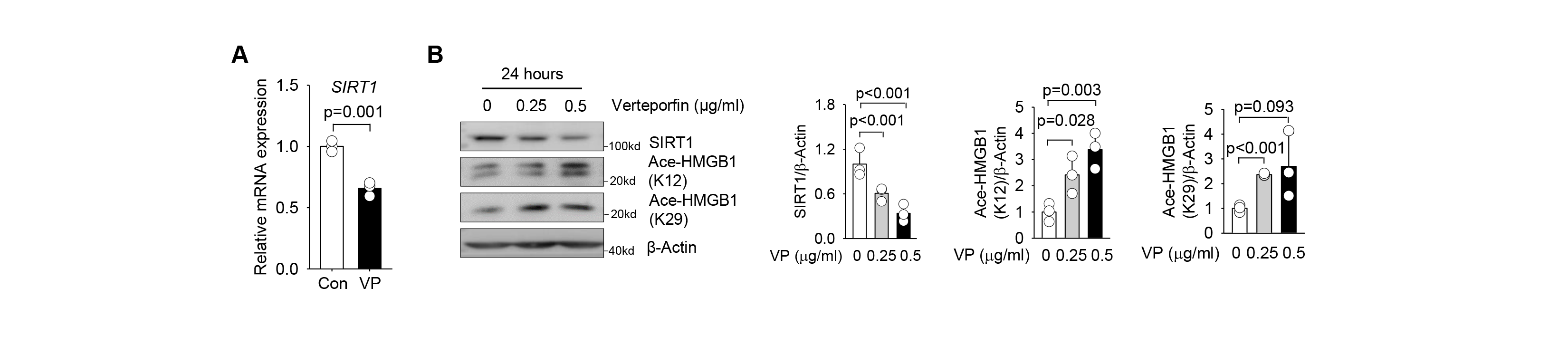

Supplement: Supplementary file 8 — Supplemental Figure 8 [file 41418_2021_841_MOESM8_ESM.tif]

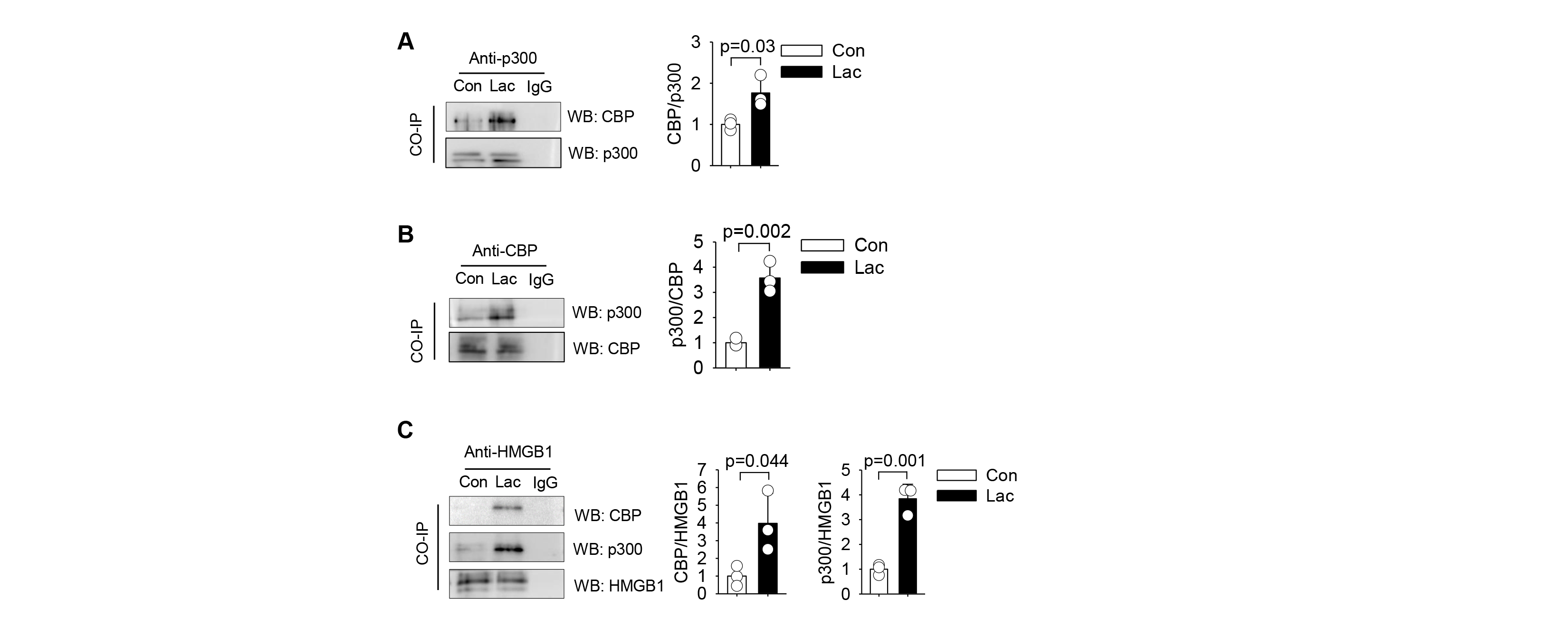

Supplement: Supplementary file 9 — Supplemental Figure 9 [file 41418_2021_841_MOESM9_ESM.tif]

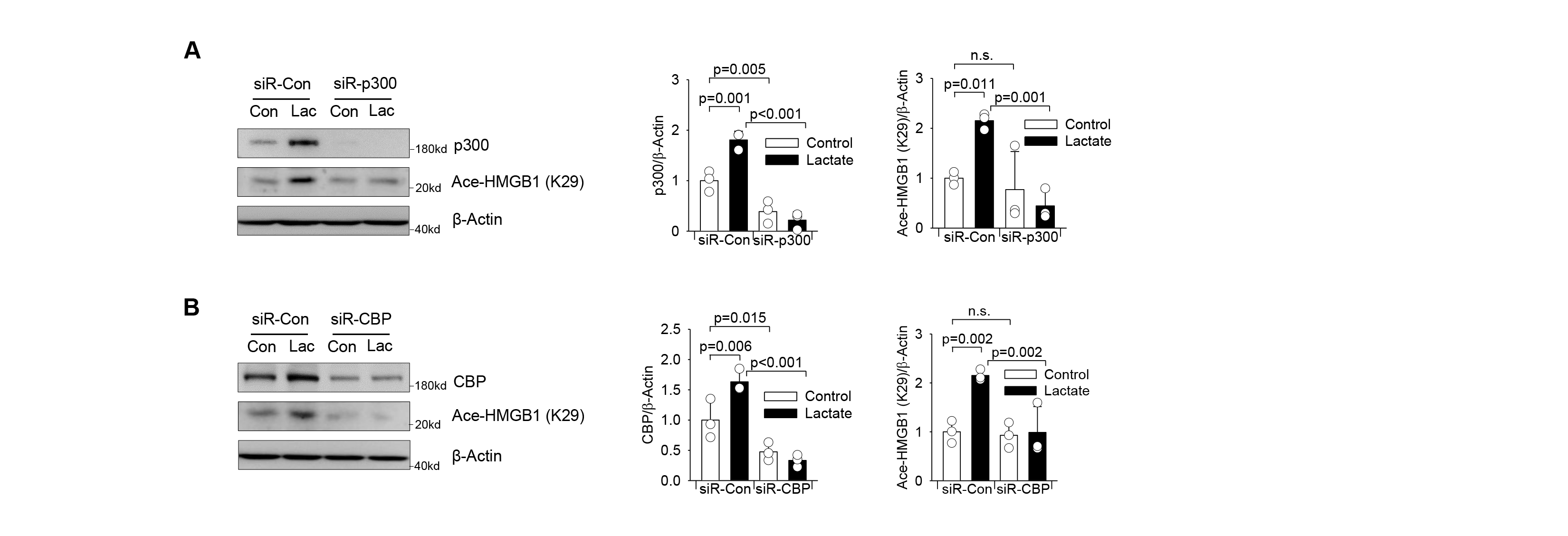

Supplement: Supplementary file 10 — Supplemental Figure 10 [file 41418_2021_841_MOESM10_ESM.tif]

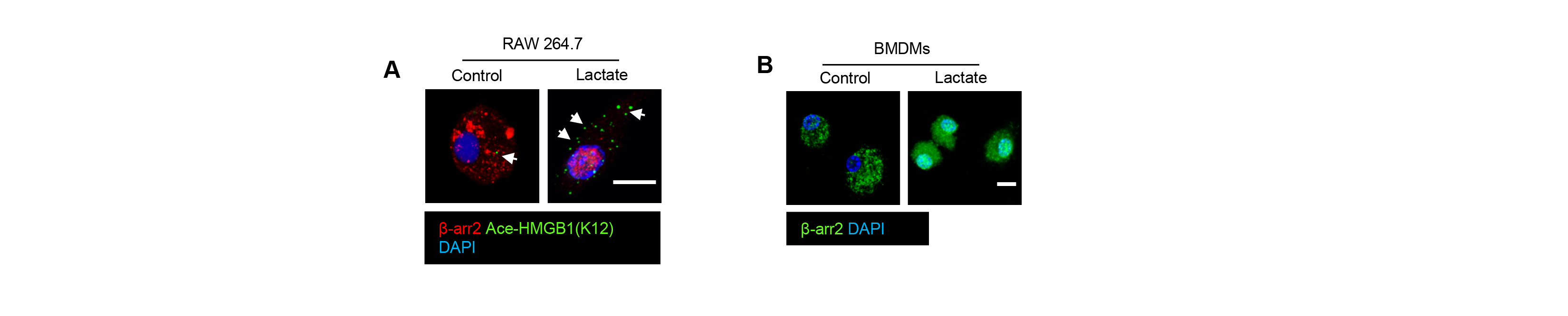

Supplement: Supplementary file 11 — Supplemental Figure 11 [file 41418_2021_841_MOESM11_ESM.tif]

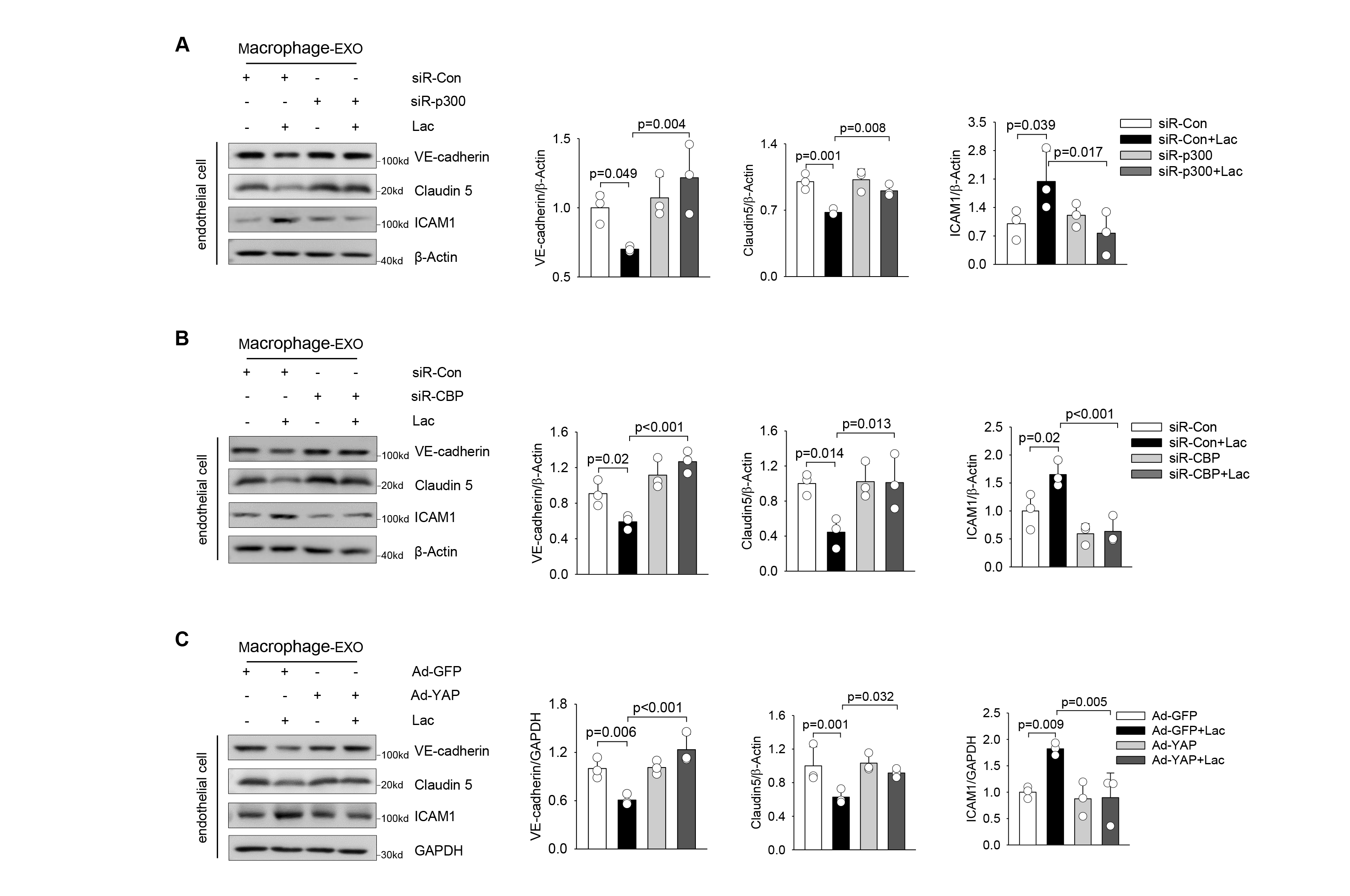

Supplement: Supplementary file 12 — Supplemental Figure 12 [file 41418_2021_841_MOESM12_ESM.tif]
