# Supplementary material for: Lactate promotes macrophage HMGB1 lactylation, acetylation, and exosomal release in polymicrobial sepsis
Source: Cell Death Differ. 2021 Aug 6;29(1):133–46. doi: 10.1038/s41418-021-00841-9 (PMC8738735; doi:10.1038/s41418-021-00841-9)
Supplement: Supplementary file 14 — Supplementary Figure legends [file 41418_2021_841_MOESM14_ESM.docx]

**Supplementary Figure Legends**

**Fig. S1. Exosome characterization.** (**A**) Representative image of exosome size (125.02 ± 24.6 nm) measured by NanoSight NS300 (*n* = 6). (**B**) Western blot shows the presence of exosomal markers and absence of ER protein calnexin in serum derived exosomes. TCL, tall cell lysate.

**Fig. S2. Cell death and apoptosis analysis.** Flowcytometry analysis of cell death (PI positive staining) and apoptosis (Annexin V positive staining) in RAW 264.7 macrophages treated with different concentrations of lactate for up to 24 hours.

**Fig. S3. Macrophages uptake extracellular lactate through MCTs for nuclear protein lactylation.** (**A**) Lactate (10 mM) induced Klac of nuclear proteins in RAW264.7 cells (*n* = 5, *t* test). (**B**) Hypoxia for 24 hours increased Klac of nuclear proteins in RAW264.7 cells, as compared with normoxia condition (*n* = 3, *t* test). (**C**) RAW 264.7 cells were treated with CHC (3 mM) or vehicle for 2 hours followed by lactate treatment for 24 hours. Total Klac levels were examined by western blot (*n* = 4 for each group, two-way ANOVA with Tukey’s test).

**Fig. S4. Lactate promotes HMGB1 acetylation and accumulation in lysosomal organelles.** Representative images of immunofluorescent staining shows lactate-induced acetylated-HMGB1 (green, indicated by white arrows) co-localized with Lamp1 (red, indicated by blue arrows) in RAW 264.7 cells. The nucleus was stained with DAPI (blue).

**Fig. S5. Lactate decreases nuclear expression of YAP and SIRT1.** Immunofluorescent staining with anti-YAP (green) and anti-SIRT1 (red) shows that lactate decreased YAP and SIRT expression and co-localization in the nucleus in RAW 264.7 cells. Co-localization analysis was performed using Zeiss Zen microscope software.

**Fig. S6. Confirmation of YAP knockout in primary peritoneal macrophages.** (**A**) Macrophage-specific YAP deficient mice were generated by crossbreeding YAP knockout mice (*YAP^fl/fl^*), in which the exon 2 of the YAP gene was flanked by loxP sites, with lysozyme2-Cre (*Lyz2-Cre^+^*) mice, in which Cre is driven by a myeloid cell-specific promoter. (**B**) Peritoneal macrophages were isolated from wild type (WT) and macrophage-specific YAP (*YAP^-/-^*) knockout mice. Peritoneal macrophage population was determined by flow cytometry using anti-CD11b and anti-F4/80 antibodies. (**C**) Immunofluorescent staining confirms the knockout of YAP (green) in peritoneal macrophages (F4/80, red).

**Fig. S7. Overexpression of YAP upregulates SIRT1 expression and decreases HMGB1 acetylation in macrophages.** Overexpression of YAP increases SIRT1 expression and decreases HMGB1 acetylation levels in macrophages. RAW 264.7 cells were transduced with Ad-GFP or Ad-YAP for 24 hours. Cytosol expression of YAP, acetylated-HMGB1 (K12), acetylated-HMGB1 (K29) and nuclear expression of YAP and SIRT1 were measured by western blot analysis (*n* = 3 for each group, *t* test).

**Fig. S8.** **Suppression of YAP transcription activity reduces SIRT1 expression and promotes HMGB1 acetylation.** (**A**) Verteporfin (VP, 0.5 µg/ml) treatment for 24 hours suppressed SIRT1 mRNA expression in RAW 264.7 cells (*n* = 3, *t* test). (**B**) VP suppressed SIRT1 protein levels and increased levels of acetylated-HMGB1 (K12) and HMGB1 (K29) in a dose dependent manner (*n* = 3, one-way ANOVA with Tukey’s test). VP, verteporfin.

**Fig. S9. Lactate induces the interaction among p300, CBP and HMGB1 in macrophages.** (**A** and **B**) Lactate-induced interaction between p300 and CBP was assayed by immunoprecipitations using anti-p300 (B) or anti-CBP (C) antibodies followed by immunoblotting with anti-CBP (B) and anti-p300 (C) antibodies, respectively. (**C**) Lactate-induced interaction between p300/CBP and HMGB1 was evidenced by immunoprecipitation with anti-HMGB1 antibodies followed by immunoblotting using anti-p300 and anti-CBP antibodies. Values are mean ± SD*. n* = 3 for each group, *t* test.

**Fig. S10. Knockdown p300 and CBP expressions by specific siRNAs attenuates lactate-induced HMGB1 acetylation in macrophages.** RAW 264.7 cells were transfected either siRNA-p300 (A) or siRNA-CBP (B) for 24 hours followed by lactate (10 mM) stimulation for 6 hours. Expression of acetylated-HMGB1 (K29) was assessed by western blot (*n* = 3 for each group, two-way ANOVA with Tukey’s test).

**Fig. S11. Lactate induces β-arrestin2 nuclear translocation and HMGB1 acetylation.** (**A**) Representative images of immunofluorescent staining using anti-β-arrestin2 (red) and anti-acetylated HMGB1 (green) show that lactate induced β-arrestin2 nuclear translocation and increased HMGB1 acetylation (indicated by white arrows). (**B**) Lactate induced β-arrestin2 (green) nuclear translocation in primary BMDMs.

**Fig. S12. Knockdown of p300 and CBP by siRNA transfection or adenovirus-mediated expression of YAP protect endothelial cell against macrophage exosome-induced downregulation of junctional proteins and upregulation of ICAM1.** (**A-B**) RAW 264.7 cells were transfected with either p300 (A) or CBP (B) siRNAs or control siRNA for 24 hours followed by lactate stimulation. (**C**) RAW 264.7 cells were transduced with Ad-YAP or Ad-GFP for 24 hours followed by lactate stimulation. Exosomes were collected from supernatants and added to endothelial cell culture. Expression of endothelial VE-cadherin, claudin 5 and ICAM1 was assayed by western blot (*n* = 3 for each group, two-way ANOVA with Tukey’s test).

**Table S1. Antibodies and reagents.**
